# Do nonpharmacological interventions prevent cognitive decline? a systematic review and meta-analysis

**DOI:** 10.1038/s41398-020-0690-4

**Published:** 2020-01-21

**Authors:** Shuqi Yao, Yun Liu, Xiaoyan Zheng, Yu Zhang, Shuai Cui, Chunzhi Tang, Liming Lu, Nenggui Xu

**Affiliations:** 1 South China Research Center for Acupuncture and Moxibustion, Medical College of Acu-Moxi and Rehabilitation, Guangzhou University of Chinese Medicine, Guangzhou, 510006 Guangdong Province P. R. China; 2 Clinical Research Center, South China Research Center for Acupuncture and Moxibustion, Medical College of Acu-Moxi and Rehabilitation, Guangzhou University of Chinese Medicine, Guangzhou, 510006 Guangdong Province P. R. China

**Keywords:** Diseases, Psychology

## Abstract

At present, prevention is particularly important when there is no effective treatment for cognitive decline. Since the adverse events of pharmacological interventions counterbalance the benefits, nonpharmacological approaches seem desirable to prevent cognitive decline. To our knowledge, no meta-analyses have been published on nonpharmacological interventions preventing cognitive decline. To investigate whether nonpharmacological interventions play a role in preventing cognitive decline among older people, we searched related trials up to March 31, 2019, in MEDLINE, EMBASE, Cochrane Central Register of Controlled Trials (CENTRAL), ClinicalTrials and the Cochrane library databases. Randomized controlled trials (RCTs) were included if they enrolled participants older than 60 years of age who had a risk of cognitive decline, and the interventions were nonpharmacological. Two reviewers independently extracted data and assessed study quality. The Grading of Recommendations Assessment Development and Evaluation (GRADE) approach was used to rate the quality of evidence. Heterogeneity was quantified with *I*^2^. Subgroup analysis and meta-regression were used to research the sources of heterogeneity. Influence analyses were used to detect and remove extreme effect sizes (outliers) in our meta-analysis. Publication bias was assessed with funnel plots and Egger test. Primary outcomes were the incidence of mild cognitive impairment (MCI) or dementia and Alzheimer’s Disease Assessment Scale–Cognitive Subscale (ADAS-Cog) scores. Second outcomes were activities of daily living (ADL) and Mini-Mental State Examination (MMSE) scores. A total of 22 studies with 13,264 participants were identified for analysis. In terms of prevention, nonpharmacological interventions appeared to be more effective than control conditions, as assessed by the incidence of MCI or dementia (RR, 0.73; CI, 0.55–0.96; moderate-certainty evidence), while the results of ADAS-Cog suggested no significant differences between two groups (MD, −0.69; CI, −1.52–0.14; very low-certainty evidence). Second outcomes revealed a significant improvement from nonpharmacological interventions versus control in terms of the change in ADL (MD, 0.73; CI, 0.65–0.80) and MMSE scores (posttreatment scores: MD, 0.25; CI, 0.02–0.47; difference scores: MD, 0.59, CI, 0.29–0.88). Univariable meta-regression showed association between lower case of MCI or dementia and two subgroup factors (*P* = 0.013 for sample size; *P* = 0.037 for area). Multiple meta-regression suggested that these four subgroup factors were not associated with decreased incidence of MCI (*P* > 0.05 for interaction). The Naive RR estimate was calculated as 0.73. When the three studies that detected by outlier and influence analysis were left out, the Robust RR was 0.66. In conclusion, nonpharmacological therapy could have an indicative role in reducing the case of MCI or dementia. However, given the heterogeneity of the included RCTs, more preregistered trials are needed that explicitly examine the association between nonpharmacological therapy and cognitive decline prevention, and consider relevant moderators.

## Introduction

Older people are at risk for cognitive decline, a condition that accompanies increases in age. Cognitive decline is usually not pathological but rather parallels a number of decreases in certain cognitive abilities^1^. It has the potential to develop into mild cognitive impairment (MCI) and eventually dementia^2^. MCI is often defined as the “symptomatic pre-dementia stage” on the continuum of cognitive decline, characterized by objective impairment in cognition that is not severe enough to require help with usual activities of daily living (ADL). It may occur as a transitional stage between normal aging and dementia. Concerns related to cognitive decline commonly manifest in healthy older adults^3^; cognitive decline has been estimated to appear in ~25–50% of community-dwelling older adults^4–6^. Evidence suggests that the performance of complex, cognitively focused daily activities may be affected by cognitive decline^7^, which may have a significant impact on the daily life of older adults and their families. Unfortunately, cognitive decline is often underestimated until there is a final progression to dementia. With the aging population, dementia is now one of the leading challenges for healthcare systems and is one of the foremost health concerns of the general public^8^; additionally, the number of people living with dementia is predicted to double by 2030^9^. The global cost has been risen to over 1 trillion dollars in 2018^10^, which is higher than the costs of cancer and cardiovascular disease combined^11^. All of the issues caused by cognitive decline have forced humans to take measures to deal with the predicament.

Acetylcholinesterase inhibitors (ACIs) have been approved by the US Food and Drug Administration (FDA) for the treatment of mild-to-moderate dementia^12^; ACIs have been accompanied by reports of adverse events, including nausea, vomiting, diarrhea, and anorexia^13,14^. Nevertheless, no FDA-approved drugs exist as therapeutic options to treat cognitive decline. By the time pharmacological interventions are necessary for dementia, the opportunity to intervene on cognitive decline has passed. Thus, the therapeutic actions associated with treating dementia are not viewed as the optimal choice. The current preventive measures for cognitive decline have been slowly realized. As a promising prevention strategy, nonpharmacological interventions preventing decline in cognitive function in older adults that do not produce adverse side effects are likely to be easier to implement and are more popular in older populations.

Given the benefits of nonpharmacological interventions, recent efforts have focused on the study of these interventions. There is no consistent evidence of a benefit for any pharmacologic agent in preventing cognitive decline in healthy older adults^15^. High-quality clinical trials have demonstrated that nonpharmacological methods could improve or maintain cognitive abilities^16,17^. Although Rodakowski et al.^6^ explored the effect of nonpharmacological interventions in slowing decline in older adults with MCI or early-stage dementia, they did not assess the at-risk population of elderly people. Therefore, the aim of the present study was to investigate whether nonpharmacological interventions play a role in preventing cognitive decline among older people.

## Methods

### Data sources and searches

We searched the MEDLINE, EMBASE and the Cochrane library databases. The last search for all databases was updated through 31 March 2019. The key words that were searched included cognitive decline, cognitive impairment, cognitive dysfunction, exercise, cognitive therapy and some other nonpharmacological methods. In addition, the ongoing trials were searched in the CENTRAL and ClinicalTrials. The detailed search strategy is shown in Supplement S1.

### Study selection

Relevant clinical trials were included if the following criteria were met: 1) randomized controlled trials (RCTs); 2) older than 60 years of age who had a risk of cognitive decline; 3) nonpharmacological interventions; and 4) at least one primary or secondary outcome was reported. Trials were excluded if they 1) were duplicate publications; 2) had participants diagnosed with MCI, Alzheimer’s disease or dementia for the aim of prevention rather than treatment; 3) were animal studies; or 4) reported insufficient information on the outcomes of interset.

### Data extraction and quality assessment

After initially browsing the titles and abstracts from the electronic databases, the full-text content of all potentially eligible studies was downloaded and analyzed. Relevant data such as the title, first author, publication year, study design, intervention for each group, outcomes and dropouts were independently extracted by two reviewers (Yao SQ and Liu Y) using inclusion criteria. Disagreements were resolved by discussion between the two reviewers and by seeking the opinion of the third author (Lu LM) if necessary.

The quality of all studies included in this review was independently evaluated by two reviewers (Yao SQ and Liu Y) using the Cochrane Collaboration’s tool^18,19^. This instrument consisted of 6 domains: Was the allocation sequence adequately generated? Was the allocation adequately concealed? Blinding:Was knowledge of the allocated interventions adequately prevented? Was loss to follow-up(missing outcome data) infrequent? Are reports of the study free of selective outcome reporting? Was the study apparently free of other problems that could put it at a risk of bias (ROB)? The tool ranks the evidence from research studies as having “high (definitely yes)”, “low (definitely no)”, “probably yes” or “probably no” levels of bias. Every study was assessed by two reviewers independently. Any disagreement was resolved by discussion with the third author (Lu LM). Certainty of evidence and strength of recommendations were evaluated using the GRADE criteria to rate confidence in summary estimates. The GRADE approach considers evidence of very low, low, moderate, and high for outcomes.

### Outcomes

The primary outcomes were the incidence of MCI or dementia and ADAS-Cog. The minimal important difference (MID) for ADAS-Cog has been reported as a 3.1 to 3.8 point reduction^20^. The diagnosis of MCI or dementia was summarized in Table S1. The ADAS-Cog consists of 11 parts and takes approximately 30 minutes to administer, which was developed as a two-part scale: one that measured cognitive functions and one that measured non-cognitive functions such as mood and behavior. The second outcomes were ADL and MMSE. The mean survey MCID for the MMSE was 3.72 (95% confidence interval 3.50–3.95) points^21^. There are six basic ADLs: eating, bathing, getting dressed, toileting, transferring and continence. The scale of MMSE included the following seven dimensions: time orientation, location orientation, immediate memory, attention and computational power, delayed memory, language, and visual space. The total score on the scale ranged from 0–30 points, with higher scores indicating better cognitive functioning. In addition, Montreal Cognitive Assessment (MoCA) and Geriatric Depression Scale (GDS) were expected to collect related information. The adverse events in the included trials were also summarized.

### Statistical analysis

All statistical analyses were performed using Reviewer Manager Software, version 5.3 (Cochrane Collaboration, Oxford, UK) and R, version 3.5.3. The overall effect was calculated using a random-effect model. Relative risk (RR) and 95% confidence intervals (CIs) were calculated for dichotomous data (e.g., the incidence of MCI and dementia). Continuous data (e.g., ADAS-Cog, MMSE scores) are presented as mean differences (MD) with 95% CIs. We reported pooled effect sizes as RR or MD with their 95% CIs and *P* values. *I*^2^ was used to evaluate the proportion of variation between studies. To explore sources of heterogeneity and better understand the effect of interventions, subgroup analysis and meta-regression models were finally fitted to investigate the sensitivity of intervention effectiveness. When the evidence was available (at least two included RCTs in one subgroup), we conducted the following pre-specified subgroup analyses: sample size (<500 and ≥500); area (America, Asia and Europe); type of intervention (cognitive training, exercise and dietary); and the duration of follow-up (<7 years and ≥7 years). We hypothesized that <500, Asia and Europe, cognitive training, <7 years had a larger effect than ≥500, America, other types of intervention and ≥7 years, respectively. Influence analyses were used to detect and remove extreme effect sizes (outliers) in our meta-analysis. The Naive (all studies included) and Robust(removed outliers) RR estimate were compared with each other. Funnel plots and Egger bias tests were used to assess publication bias.

## Results

### Study selection

**Fig. 1:**
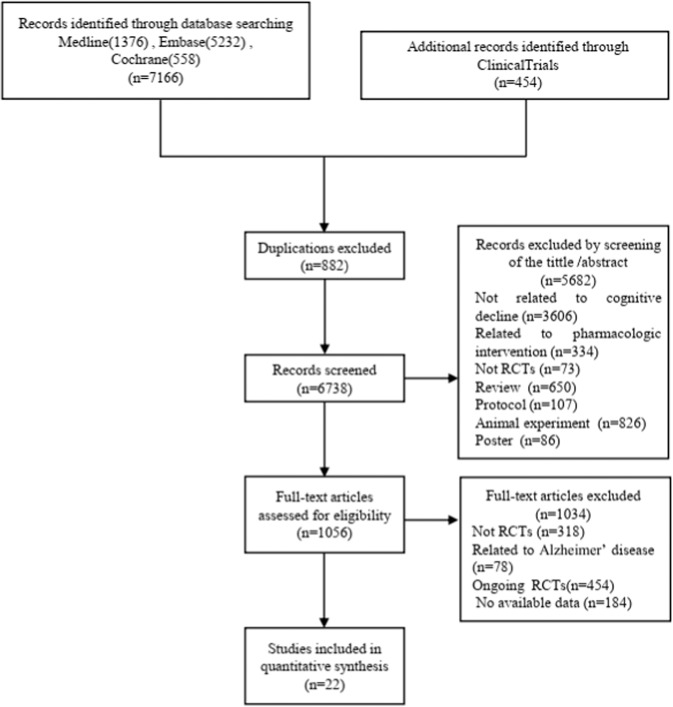
Flowchart of the trial selection process.

A total of 7620 potentially relevant citations were identified. A total of 882 duplicate papers were first removed. A total of 5682 records were excluded by scanning the titles and abstracts. After reading the full texts of the remaining articles, 1034 studies were excluded for the following reasons: not RCTs (*n* = 318), related to Alzheimer’s disease (*n* = 78), ongoing RCTs (*n* = 454) and no available data (*n* = 184). Finally, 22 articles were identified for inclusion in this research (Fig. 1). The 22 reports were published from 2006 to 2018. The language of the publications was English.

### Characteristics of the included studies

Table 1 shows the characteristics of the included studies in this review. Among the 22 included studies, all the studies were reported in English. Eight (36.4%) of the studies^22,23^ investigated the incidence of MCI or dementia. Only two of (9.1%) the studies^24,25^ reported the ADAS-Cog as an outcome. ADL was calculated in two trials (9.1%) and 15 of (68.2%) of the studies^22,26–28^ investigated the MMSE. Regarding the interventions, eight studies (39.1%)^22,24,25,29–32^ examined exercise, 10 studies (43.5%)^23,27,28,33–37^ were about diet, and four studies (18.2%)^26,38–40^ were cognitive training interventions with comparisons to a control group. All the studies reported the number of dropouts.

**Table 1:** Characteristics of included studies.

Study ID	Mean age in years	Final semple size Experimental/Control	Intervention	Follow-up	Control	Outcomes	Risk of bias
Linda^18^	77.8	92/169	Tai Chi	1 year	Stretching and toning exercise	①④	High
Lapiscina^19^	74.6	224/132	MedDiets	6.5 years	Low-fat control diet	①④	Low
Petrelli^20^	68.9	16/14	Cognitive training	1 year	Waiting list	①④	High
Kryscio^21^	67.5	1799/1830	Vitamin E	7 years	Placebo		Low
Sink^22^	70–89	743/747	Physical activity	2 years	Health education	①	Low
Edwards^33^	73.6	574/552	Cognitive training	10 years	Untreated control	①	Low
Shi^26^	63.9	81/83	Cognitive training	1 year	Medical treatment	①③	High
DeKosky^34^	79.1	1448/1429	Ginkgo biloba	6.1 years	Placebo	①	Low
Lautenschlager^29^	68.6	69/69	Exercise	18-month	Education and usual care	②	Low
Olivia^38^	72	13/13	Physical training	3-month	Wait-list control	②	High
Kwok^39^	83.2	162/183	Dietary support	2 years	Regular group dietary	④	High
Vanessa^23^	74	194/196	Docosahexaenoic acid (DHA)–rich fish oil	2 years	Olive oil	④	Low
Karin 2010^24^	70	219/218	Docosahexaenoic acid	24 weeks	Placebo	④	Low
Daniela^25^	69.6	30/30	Cocoa flavanols	8 weeks	Flavanol	④	High
McDougall^27^	75	127/117	Memory intervention	2 years	Health intervention	④	High
Simone^58^	82.3	54/57	Vitamin B-12	24 weeks	Placebo	④⑤	High
Piedra^41^	73.1	212/207	Exercise	2 years	Health education	④	High
Arnaud^35^	82.4	41/51	Tai Chi	1 year	Usual care	③④⑤	Low
Hiroyuki^40^	70.4	53/47	Golf training	24 weeks	Health education	④⑤	Low
Cinta Valls^36^	66.9	127/95	Mediterranean diet	7 years	Control diet	④	Low
Antonio^30^	69.2	53/56	Exercise training	1 year	Educational suggestions	④	High
Jagadish K^42^	75	327/311	Multi-domain intervention	3 years	Placebo	④	Low

①The icidence of MCI or dementia ②ADAS-Cog ③ADL ④MMSE ⑤GDS

### Assessment of ROB

The ROB evaluation for each included RCT is summarized in Fig. S1. All the included trials had “low risk(definitely no)” or “probably yes” levels of bias. Nineteen (86.4%) studies were at low overall ROB and 2 (9%) studies were at high overall risk because of incomplete outcome data and blinding of outcome assessment^33,41^. In addition, one studies (5%) at high overall risk due to incomplete outcome data and blinding of participants^22^. Agreement between reviewers was substantial for 6 domains and perfect for 1 domain.

### Primary outcomes

#### The incidence of MCI or dementia and ADAS-Cog

**Fig. 2:**
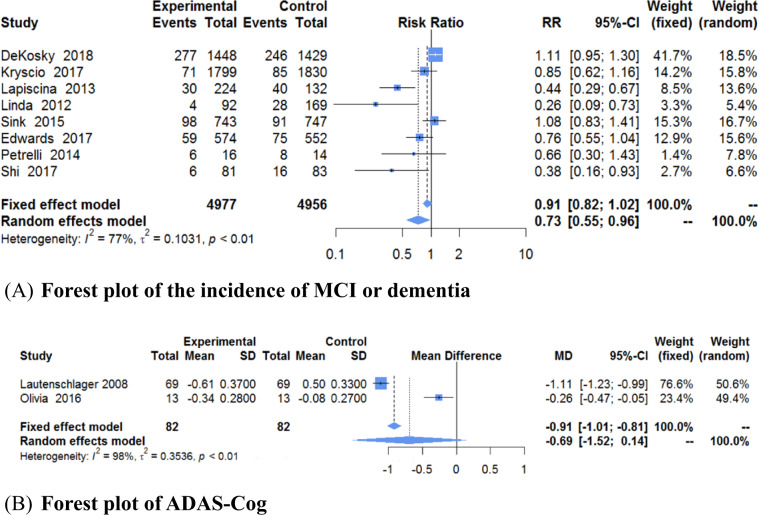
Forest plot of the incidence of MCI or dementia and ADAS-Cog. **a** Forest plot of the incidence of MCI or dementia and **b** forest plot of ADAS-Cog.

The results of the incidence of MCI or dementia, are shown in Fig. 2a. There were 8 studies^22,23^ with 9933 old adults that were evaluated for this outcome. There were 4977 participants in the experimental groups and 4956 in the control groups. A heterogeneity test (*χ*^2^ = 30.74, *P* < 0.0001, *I*^2^ = 77%) indicated that there was clear statistical heterogeneity between studies. Thus, a random-effect model was used to calculate the combined RR and 95% CI (RR = 0.73, 95% CI, 0.55 to 0.96, *P* = 0.03). When RR was 0.73, 95% CI, 0.55–0.96, it means that compared to control, the nonpharmacological interventions decreased the incidence of MCI or dementia by 27%, with a 45% decrease on one side and a 4% decrease on the other side of 95% interval. The results of the meta-analysis showed that there was a statistically significant difference in the incidence of MCI or dementia between the experimental and control groups. In addition, ADAS-Cog was only reported in 2 trials (Fig. 2b) and there was no significant difference between two groups (MD = −0.69, 95% CI, −1.52 to 0.14, *P* = 0.10). When MD was −0.69, 95% CI, −1.52 to 0.14, the score of ADAS-Cog decreased by 0.69, with a 1.52 decrease on one side and a 0.14 increase on the side of 95% interval.

### Second outcomes

#### ADL and MMSE

**Fig. 3:**
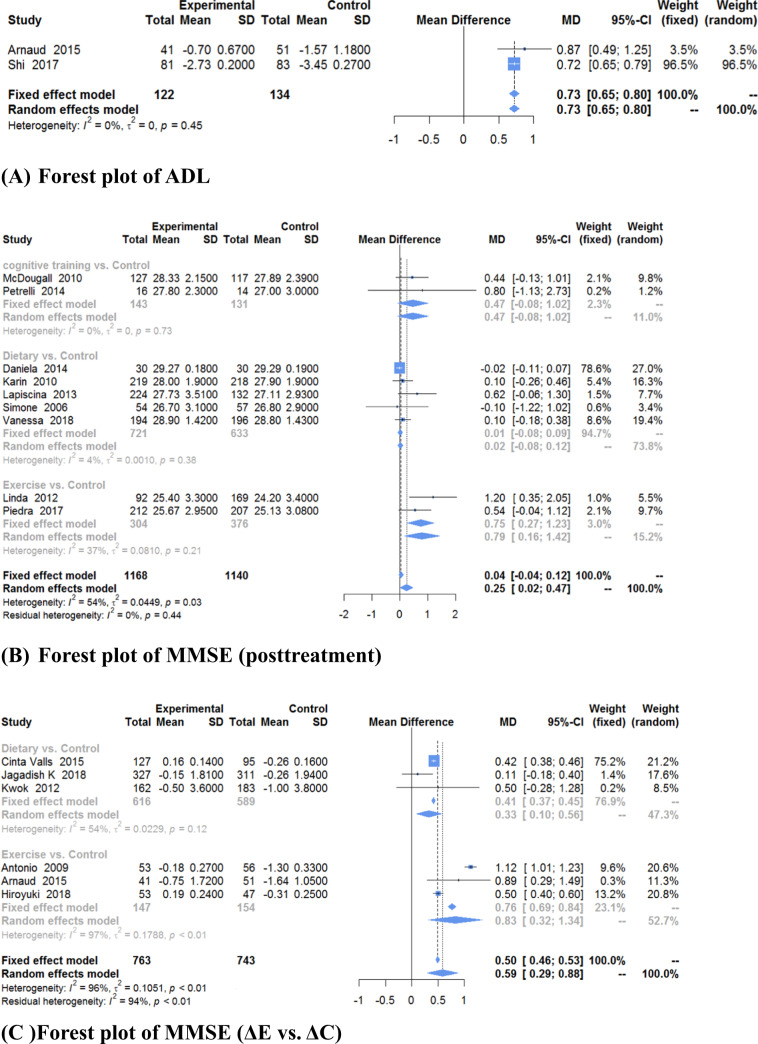
Forest plots of ADL and MMSE scores (different types of intervention). **a** Forest plot of ADL. **b** Forest plot of posttreatment MMSE scores. **c** Forest plot of MMSE difference scores (ΔE vs. ΔC). ΔE: The mean difference before and after treatment in the experimental group. ΔC: The mean difference before and after treatment in the control group.

The results of ADL and MMSE scores, are shown in Fig. 3. Only 2 trials^39,42^ with 256 old adults evaluated the ADL. Fixed-effect model was used to calculate MD and 95% CI (MD = 0.73, 95% CI, 0.65–0.80, *P* < 0.00001) for the heterogeneity test (*χ*^2^ = 0.57, *P* = 0.45, *I*^2^ = 0%). As for the MMSE, there were two types of scores for the MMSE: posttreatment MMSE scores and differences in MMSE scores from before and after treatment. There were 15 trials^22,26–28^ with 3992 old adults that evaluated the MMSE. A total of 2025 participants were in the experimental groups, and 1967 were in the control groups. Random-effect model was used to calculate the MD and 95% CI (posttreatment scores: MD = 0.25, 95% CI, 0.02–0.47, *P* = 0.03; difference scores: MD = 0.59, 95% CI, 0.29–0.88, *P* < 0.0001) for the clear statistical heterogeneity between studies (posttreatment scores: *χ*^2^ = 17.27, *P* = 0.03, *I*^2^ = 54%; differences scores: *χ*^2^ = 139.30, *P* < 0.00001, *I*^2^ = 96%). The results of the meta-analysis showed that there was a statistically significant increase in ADL and MMSE scores between the experimental and control groups, indicating that nonpharmacological interventions can significantly increase ADL and MMSE scores. However, the MMSE score did not achieve the MID.

Other outcome associated with mood that can influence cognitive performance had also reported in this study. The result of GDS had been shown in Fig. S2. There was clear statistical heterogeneity between the 3 studies (*χ*^2^ = 18.22, *P* = 0.0001, *I*^2^ = 89%). Thus, random-effect model was used to calculate the MD and 95% CI, (MD = −0.49, 95% CI, −1.08 to 0.09, *P* = 0.10). However, MoCA was failed to summaried for the lack of related data.

### Subgroup analysis and meta-regressions

The subgroups were divided into small groups according to sample size, the area of RCT, the type of intervention or the duration of follow-up (Table 2). Univariable meta-regression showed association between lower case of MCI or dementia and two subgroup factors (*P* = 0.013 for sample size; *P* = 0.037 for area). Another two subgroup factors didn’t show difference (*P* = 0.621 for intervention; *P* = 0.739 for follow-up). Multiple meta-regression suggested that these four subgroup factors were not associated with decreased incidence of MCI (*P* > 0.05 for interaction). However, the heterogeneity was significantly decreased after the analysis of subgroups (Fig. S3). In the subgroups divided by the number of sample, the source of heterogeneity could be the studies with ≥500 sample size (≥500: *I*^2^ = 50%; <500: *I*^2^ = 0%). In the area of RCTs, the result of forest plot showed the trials in Asia and Europe had no heterogeneity (Asia: *I*^2^ = 0%; Europe: *I*^2^ = 0%), while in America, the value of *I*^2^ was 35%. When considered the different type of nonpharmacological interventions, cognitive training had obvious significance (*I*^2^ = 0%, *P* = 0.01), compared with exercise and dietary (exercise: *I*^2^ = 86%, *P* = 0.45; dietary; *I*^2^ = 88%, *P* = 0.29). The duration of follow-up was also a factor of subgroup. There was no heterogeneity in the trials that the follow-up was more than 7 years (≥7 years: *I*^2^ = 0%; <7 years: *I*^2^ = 82%). In summary, the results of subgroups suggested that the heterogeneity in this study might originate from the sample size, type of intervention and the duration of follow-up.

**Table 2:**
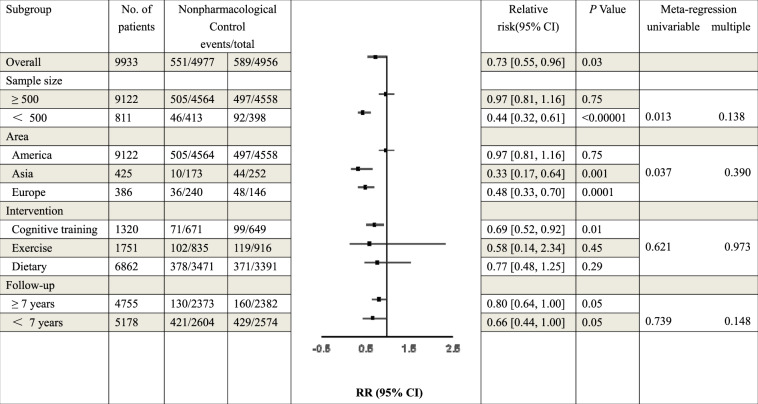
Results of subgroup analysis and meta-regression.

### Grading of recommendations assessment, development and evaluation (GRADE)

The Table S2 summarized the certainty of evidence and strength of recommendations. For cognitively normal adults, very low-certainty evidence suggests that nonpharmacological interventions have little effect on the prevention of cognitive decline for the result of ADAS-Cog. Meanwhile, moderate-certainty evidence of the incidence of MCI or dementia suggested that the older in intervention group may associated with lower incidence of MCI or dementia, compared with control. The quality of second outcomes, ADL, MMSE (posttreatment scores), MMSE (difference scores), GDS, were assessed as low-certainty, low-certainty, moderate-certainty and very low-certainty evidence, respectively.

### Meta-analysis of prevention acceptability

The result of the prevention acceptability was shown in Fig. S4, which showed prevention-related loss to follow-up had no statistically significant difference when participants received nonpharmacological interventions compared with controls (RR = 0.95, 95% CI, 0.81–1.10, *P* = 0.46).

### Outlier and influence analysis

The results of outlier and influence analysis in the incidence of MCI or dementia showed the three^23,29,33^ studies were probably outliers which may distort the effect size estimate in the nonpharmacological interventions. The Baujat Plot and Leave-One-Out-Analyses were present in the Fig. S5.

### Publication bias and robust estimate

The results of funnel plot and Egger bias tests were shown in Fig. S6. The Naive RR estimate was calculated as 0.73 (*I*^2^ = 77%, *P* = 0.03). When the 3 studies that detected by outlier and influence analysis were left out, the Robust RR was 0.66 (*I*^2^ = 43%, *P* = 0.01). Egger bias tests for the incidence of MCI or dementia (bias = 0.24; 95% CI, –1.77 to 5.74) revealed that publication bias could be not exist.

### Adverse events

There were no severe adverse events adjudicated to be related to the interventions in either group during the study period in 9 trials and 11 trials had not reported the adverse events. Only two reports had obvious adverse events. In the study of dietary intake about *Ginkgo biloba*, the adverse event profiles for *Ginkgo biloba* and placebo were similar and there were no statistically significant differences in the rate of serious adverse events^23^. Although another about physical activity reported ten events occurred during the study and intervention staff judged that it was unlikely any of these events were directly caused by the intervention^24^.

## Discussion

### Summary of main findings

The present review comprised 22 RCTs with 13,264 patients from three databases published in English. Our findings provide evidence that nonpharmacological interventions couldn’t prevent cognitive decline for the result of ADAS-Cog (very low-strength evidence) showed no significant difference between nonpharmacological interventions and controls. However, nonpharmacological therapy could have an indicative role in reducing the case of MCI or dementia that supported by the outcome of the incidence of MCI or dementia (moderate-strength evidence). Meanwhile, the results of second outcomes suggested that nonpharmacological interventions could be beneficial in improving the ADL and MMSE scores. Although evidence for prevention of cognitive decline was insufficient, nonpharmacological therapy could have an indicative role in reducing the case of MCI or dementia.

### Applicability of the current evidence

In clinical practice guidelines for dementia in Australia^43^, a timely diagnosis has been emphasized, along with the use of nonpharmacological approaches as early as possible. Furthermore, nonpharmacological interventions should be implemented before considering the use of medications. However, there is still a lack of guidelines for the specific prevention of cognitive decline. Based on our results, nonpharmacological interventions were determined to show little influence on the ADAS-Cog, but exactly have a certain influence on the incidence of MCI or dementia in the follow-up.

As for the type of nonpharmacological interventions, physical exercise, dietary and cognitive training were categorized in this study. Exercise had been reported as an effective intervention on cognitive function. Smith et al.^44^ performed a meta-analysis on the relationship between aerobic exercise and neurocognitive performance. In their meta-analytic review of 29 studies, individuals in the aerobic exercise training group demonstrated modest improvements in neurocognitive performance. In present study, exercise seemed to be no power on the prevention of cognitive decline. These inconsistent conclusions may be the reasons of different outcomes and perspective.

As a form of nonpharmacological intervention, dietary recommendations, such as the MedDiet, have always been a focus. Several studies have suggested beneficial changes in cognitive function after dietary interventions^16,45,46^ and the MedDiet has been associated with slower cognitive decline and a lower risk of developing AD^47,48^. However, the latest research showed that the MedDiet did not improve cognitive function in the Australian sample^49^, which is consistent with our results showing that dietary interventions had little protective effect against cognitive function that is declining or becoming impaired.

Cognitive training is usually discussed as an manner of mental exercise, which focuses on guided practice on a set of tasks that reflect particular cognitive functions, such as memory, attention or problem-solving. In addition, the effect of cognitive training on the cognitive ability had been illustrated to be controversial. Naqui et al concluded that cognitive training might have a benefit in preventing cognitive decline according to 3 RCTs^50^, especially in the performance of reasoning, speed, and memory. The currently used method of preventing cognitive decline in older adults was elucidated in the report of Brodziak et al.^15^, which is a guideline for the prevention and treatment of cognitive impairment in the elderly. The research involved not only pharmacological interventions but also nonpharmacological interventions that mainly consisted of physical exercise and cognitive training in preventing cognitive decline; these findings were partly supported by the results from the present study, which suggested that cognitive training had the role of prevention in the incidence of MCI or dementia, which prompted a potential maner to be good for the cognitive ability. Although it promoted to slow or prevent cognitive decline, including dementia^51^, there are still reports doubt the effectiveness. In recent reviews, evidence regarding prevention or delay of cognitive decline or dementia is insufficient^52,53^.

Though the primary outcomes in this study hadn’t provided robust evidence to suggest that nonpharmacological interventions could prevent cognitive decline, the effect of nonpharmacological interventions on the incidence of MCI or dementia could be a clue to decrease the case of MCI or dementia in the follow-up. Perhaps more important, is the unanticipated benefits of being in an intervention group with several aspects of an intervention that are psychosocial having a global effect on mood, social contact and confidence that can influence cognitive performance but may have little to do with brain pathophysiology and subsequent decline that truly represents neuronal cell death. In present study, the results of ADL and MMSE scores demonstrated nonpharmacological interventions exactly have a good influence. However, only two trials reported ADL in their studies. More high-quality researches are needed to validate. MMSE is the most commonly used brief cognitive tool in the assessment of a variety of cognitive disorders. Though MMSE does not perform well as a confirmatory (case-finding) tool for dementia, MCI, and delirium, but it does perform adequately in a rule-out (screening) capacity. For those scoring below threshold on MMSE, a more extensive neuropsychological and clinical evaluation should be pursued.

Though the subgroup analysis suggested the potential source of heterogeneity. It seemed that the sample size, area of RCTs, type of nonpharmacological interventions and the duration of follow-up could be the influencing factor. The heterogeneity was reduced after the analysis of subgroups. In our univariable meta-regression, sample size and the area of RCT could be associated with lower incidence of MCI or dementia. However, the result of multiple meta-regression suggested no differential effects among the four prespecified hypotheses. After checking against 11 criteria^54,55^ for assessing the credibility of an apparent subgroup effect, we thus concluded that the subgroup hypothesis has low credibility.

Since the value brought about by pharmacological interventions has been very limited and has been accompanied by clear side effects^56,57^, the need for identifying potential prevention measures has transitioned from being necessary to urgent. Taken together, none of the included studies reported significant or serious adverse events brought by the nonpharmacological interventions. The included studies addressed the safety of nonpharmacological interventions. As for the effectiveness, current evidence suggested the effectiveness of nonpharmacological interventions was insufficient. But, these nature methods did reduce the incidence of MCI or dementia, which implied a potential manner for the related researches in the future. Therefore, nonpharmacological interventions would be benefit for decreasing the incidence of MCI or dementia and the life quality in the older.

### Limitations of this review

There are some limitations to this review. First, ADAS-Cog, as an primary outcome of this study (very low-certainty evidence), had very limit evidence to confirm the prevention of nonpharmacological methods because the included studies were inadequate. Second, despite of the significant differences between nonpharmacological interventions and controls, the extent to decrease of incidence of MCI or dementia brought by nonpharmacological interventions remains unknown. In addition, the outcomes we have collected were limited and involved only the incidence of MCI or dementia, ADAS-Cog, ADL and MMSE scores. Other outcome, such as the MoCA, which has a sensitivity of 80–100% and specificity of 50–76% for detecting MCI, was not included in the analysis due to the lack of relevant information. Finally, the conclusion of this present review was limited by the quality and number of the studies included in the analysis.

## Conclusions

Current evidence for prevention of cognitive decline was insufficient. Nonpharmacological therapy could have an indicative role in reducing the case of MCI or dementia. This meta-analysis provides important new evidence that nonpharmacological therapy may be effective in reducing the case of MCI or dementia among older people. For acceptability, nonpharmacological therapy was not significantly associated with fewer dropouts than control groups. Given the heterogeneity of the included RCTs, we call for large, preregistered RCTs of good quality investigating the association between nonpharmacological therapy and cognitive decline prevention, and considering relevant moderators.

## Supplementary information

Supplementary information

Supplement S1

Fig. S1

Fig. S2

Fig. S3

Fig. S4

Fig. S5

Fig. S6

Table S1

Table S2
