# Supplementary material for: Do nonpharmacological interventions prevent cognitive decline? a systematic review and meta-analysis
Source: Transl Psychiatry. 2020 Jan 21;10:19. doi: 10.1038/s41398-020-0690-4 (PMC7026127; doi:10.1038/s41398-020-0690-4)
Supplement: Supplementary file 1 — Supplementary information [file 41398_2020_690_MOESM1_ESM.doc]

**Supplement S1** Search strategy for each database

**Table S1**  Diagnosis of MCI or dementia in the included trials

**Fig. S1** Assessment of risk of bias

**Fig. S2** Forest plot of GDS

**Table S2** Summary of strength of evidence for outcomes

**Fig. S3** Forest plot of subgroups

**Fig. S4** Forest plot of prevention acceptability

**Fig. S5** Outlier and influence analysis

**Fig. S6** Funnel plot and Egger test

**Supplement S1** Search strategy for each database

The search was originally carried out on March 31, 2019.

Databases: Medline, EMBASE, Cochrane library, CENTRAL, ClinicalTrials database

| Database | Search | Included Returns |
| --- | --- | --- |
| EMBASE/  MEDLINE | #1. cognitive decline.mp. or Cognitive Dysfunction/  #2. cognitive impairment.mp. or Cognitive Dysfunction/  #3. Cognitive Dissonance/  #4. (Cognitive Impairment or Cognitive Impairments or Neurocognitive Disorder or Decline, #Cognitive or Mental Deteriorations or Mental Deterioration or Declines, Cognitive or #Cognitive Declines or Cognitive Decline or Deterioration, Mental or Deteriorations, Mental or Neurocognitive Disorders or Dissonance, Cognitive or Dissonances, Cognitive or Cognitive Dissonances or Cognitive Aging).mp.  #5: #1 or #2 or #3 or #4  #6. exp Exercise/ OR exp Exercise Therapy/ OR exp "Physical Education and Training"/ OR Physical Fitness/ OR Physical Exertion/ OR exp Walking/ OR Running/ or Jogging/ OR Swimming/ OR (cycling or bicycling).tw. OR (exercise$ or exercising).tw. OR (physical adj3 (education or training)).tw. OR exp physical activity/ OR exp sport/ OR exp Combined Modality Therapy/ OR Exercise/ OR Physical Exertion/ OR exp Physical Therapy Modalities/ OR (alexander adj (technique or method)).tw. OR Biofeedback, Psychology/  #7. yog*.af. OR Meditation OR meditat*.af. OR Relaxation OR relax*.af. OR yoga* OR meditat* OR relaxation* OR Relaxation Therapy/  #8. hatha OR ashtanga OR bikram OR iyengar OR kripalu OR kundalini OR sivananda OR vinyasa OR raja OR radja OR bhakti OR jnana OR kriya OR karma OR yama OR niyama OR asana OR pranayama OR pratyahara OR dharana OR dhyana OR samadhi OR bandha OR mudra  #9. PLYOMETRIC EXERCISE/ or COOL-DOWN EXERCISE/ or EXERCISE/ or EXERCISE MOVEMENT TECHNIQUES/ or EXERCISE THERAPY/ or WARM-UP EXERCISE/  #10. Psychology,Social.mp. or Psychology, Social/  #11. PSYCHOTHERAPY, RATIONAL-EMOTIVE/ or PSYCHOTHERAPY/ or PSYCHOTHERAPY, MULTIPLE/ or "IMAGERY (PSYCHOTHERAPY)"/ or PSYCHOTHERAPY, GROUP/ or Psychotherapy.mp. or PSYCHOTHERAPY, BRIEF/ or PSYCHOTHERAPY, PSYCHODYNAMIC/  #12. PSYCHOTHERAP$2.tw. OR RELAX$6.tw. OR (PSYCHOLOG$5 adj4 INTERVENT$5).tw. OR Relaxation Training/ OR exp Counseling/ OR (COUNSELLING or COUNSELING).tw. OR ((BEHAVIOR$4 or BEHAVIOUR$4) adj4 (MODIFY or MODIFICAT$4 or THERAPY$2 or CHANGE)).mp. OR Stress Management/ OR ((BEHAVIOR$4 or BEHAVIOUR$4) adj4 (MODIFY or MODIFICAT$4 or THERAPY$2 or CHANGE)).tw. OR (STRESS adj3 MANAGEMENT).tw. OR exp Meditation/ OR MEDITAT$5.tw. #13. (MANAGE$5 adj2 (ANXIETY or DEPRES$5)).tw. OR CBT.tw. OR HYPNOTHERAP$2.tw. OR (GOAL$2 adj3 SETTING).tw. OR (MOTIVAT$5 adj4 INTERVENT$6).tw. OR Psychosocial Care/ OR Psychosocial Rehabilitation/ OR PSYCHOSOCIAL.tw. OR Autogenic Training/ OR AUTOGENIC.tw.  #14. COGNITIVE THERAPY/ OR BEHAVIOR THERAPY/  #15. (cogniti$ adj3 (behavio$ or intervention$ or psychotherap$ or technique$ or therap$ or treat$)).ti,ab.  #16. (behavio$ adj3 (intervention$ or psychotherapy$ or technique$ or therap$ or treat$)).ti,ab.  #17. Cognitive Therapy.mp. or Cognitive Therapy/ OR (cognit* adj2 stimulation).mp. OR (cognit* adj2 rehabilitation).mp or (cognit* adj2 training).mp. or (cognit* adj2 retrain*).mp. OR cognitive support.mp. OR memory function.mp. OR (memory adj2 rehabilitation).mp. OR (memory adj2 therap*).mp.or memory aid.mp. OR memory group.mp. OR memory training.mp. OR memory retraining.mp. OR memory support.mp OR memory stimulation.mp. OR memory management.mp.  #18.Cognitive Therapy.mp. or Cognitive Therapy/ or (cognit* adj2 stimulation).mp.or  (cognit* adj2 rehabilitation).mp or (cognit* adj2 training).mp. or (cognit* adj2 retrain*).mp. or cognitive support.mp. or memory function.mp. or (memory adj2 rehabilitation).mp. or (memory adj2 therap*).mp.or memory aid.mp. or memory group.mp. or memory training.mp. or memory retraining.mp. or memory support.mp or memory stimulation.mp. or memory management.mp.  #19.exp Electric Stimulation Therapy/ OR exp Rehabilitation/ OR Hydrotherapy/ OR postur* correction.mp. OR Feldenkrais.mp.  #20. Complementary Therapies.mp. or Complementary Therapies/  #21. (Therapies, Complementary or Therapy, Complementary or Complementary Medicine or Medicine, Complementary or Alternative Medicine or Medicine, Alternative or Alternative Therapies or Therapies, Alternative or Therapy, Alternative).mp.  #22. Taichi or Taiji or Ba duan jin  #23. (art therapy or Reminiscence therapy or Validation therapy or Simulated presence therapy or Light therapy).mp.  #24. (Animal therapy or Aromatherapy or Snoezelen room Exercise or Cognitive training and rehabilitation or group exercise or walking programs or cognitive stimulation treatment).mp.  #25. (progressive muscle relaxation or cognitive training or Physiotherapy or acupuncture or Homeopathy or Hynosis or Massage or Naturopathy or Osteopathy or relaxation).mp.  #26: #6 or #7 or #8 or #9 or #10 or #11 or #12 or #13 or #14 or #15 or #16 or #17 or #18 or #19 or #20 or #21 or #22 or #23 or #24 or #25  #27. random*.ti,ab. or factorial*.ti,ab. or (crossover* or cross over* or cross-over*).ti,ab. or placebo*.ti,ab. or (doubl* adj blind*).ti,ab. or (singl* adj blind*).ti,ab.or assign*.ti,ab. or allocat*.ti,ab. or volunteer*.ti,ab. or CROSSOVER PROCEDURE.sh. or DOUBLE-BLIND PROCEDURE.sh. or RANDOMIZED CONTROLLED TRIAL.sh. or SINGLE BLIND PROCEDURE.sh. or cohort*.ti,ab. or cohort analysis/ or prospective study/ or case*.ti,ab.  #28. Comparative Study/ or exp Evaluation Studies/ or exp Follow Up Studies/ or exp Prospective Studies/ or exp Epidemiologic Studies/ or exp Case Control Studies/ or exp Cohort Studies/ or (cohort adj (study or studies)).mp. or cohort analy$.mp. or (follow up adj (study or studies)).mp. or (observational adj (study or studies)).mp. or retrospective.mp. or prospective$.mp.  #29: #27 or #28  #30. prevention* or protect* or protective or prevent  #31: #5 and 26 and #29 and #30 | EMBASE: 5232  MEDLINE: 1376 |
| Cochrane | #1. MeSH descriptor: [cognitive decline] explode all trees  #2. MeSH descriptor: [cognitive dysfunction] explode all trees  #3. MeSH descriptor: [cognitive impairment] explode all trees  #4. MeSH descriptor: [Cognitive Dissonance] explode all trees  #5.（ Cognitive Impairment OR Cognitive Impairments OR Neurocognitive Disorder OR Decline, Cognitive OR Mental Deteriorations OR Mental Deterioration OR Declines, Cognitive OR Cognitive Declines OR Cognitive Decline OR Deterioration, Mental OR Deteriorations, Mental OR Neurocognitive Disorders OR Dissonance, Cognitive OR Dissonances, Cognitive OR Cognitive Dissonances OR Cognitive Aging ）:ti,ab,kw (Word variations have been searched)  #6: #1 or #2 or #3 or #4 or #5  #7. MeSH descriptor: [Psychology,Social.] explode all trees  #8. MeSH descriptor: [Psychotherapy] explode all trees  #9.(incentive*OR voucher OR psychotherap* OR psychosocial* OR behaviour therapy OR behavior therapy OR reinforcement OR motivation* OR contingent* OR advice OR biofeedback OR community OR stimulation OR education* OR brief intervention OR early intervention OR minimal intervention OR counseling OR counsel* OR cognitive therapy OR family therapy OR social skill OR stress management training OR supportive expressive therapy OR neurobehavioral* OR coping skill* OR "self-control training"):ti,ab,kw (Word variations have been searched)  #10. MeSH descriptor: [Complementary Therapies] explode all trees  #11. (Therapies, Complementary or Therapy, Complementary or Complementary Medicine or Medicine, Complementary or Alternative Medicine or Medicine, Alternative or Alternative Therapies or Therapies, Alternative or Therapy, Alternative):ti,ab,kw (Word variations have been searched)  #12. MeSH descriptor: [exercise] explode all trees  #13. (Yoga OR art therapy OR Reminiscence therapy OR Validation therapy OR Simulated presence therapy OR Light therapy OR Animal therapy OR Aromatherapy OR Snoezelen room Exercise OR Cognitive training and rehabilitation OR group exercise OR walking programs OR cognitive stimulation treatment OR mindfulness-based Alzheimer’s stimulation OR progressive muscle relaxation OR cognitive training OR Physiotherapy) :ti,ab,kw (Word variations have been searched)  #14. Synonyms: Exercise, Acute; Exercises, Acute; Acute Exercises; Acute Exercise; Physical Activities; Activities, Physical; Exercises; Activity, Physical; Physical Activity; Exercises, Physical; Physical Exercise; Exercise, Physical; Physical Exercises; Exercise, Aerobic; Aerobic Exercise; Exercises, Aerobic; Aerobic Exercises; Training, Exercise; Exercise Trainings; Trainings, Exercise; Exercise Training; Isometric Exercise; Exercise, Isometric; Exercises, Isometric; Isometric Exercises  #15. MeSH DESCRIPTOR Behavior Therapy Explode All  #16. MeSH DESCRIPTOR Psychotherapy  #17. CBT:TI,AB,KW  #18. cognitiv* NEAR3 (behav* or treatment* or technique* or therap* or intervention* or restructur* or reappraisal*)  #19. behav* NEAR3 (treatment* OR therap* or intervention* OR activat* or technique* or modif* or change*)  #20. coping* NEAR3 (skill* or strateg*)  #21. psychotherap* or psychological*  #22. MeSH descriptor Psychotherapy explode all trees #23. psychotherap* or psycholog* NEAR intervent* or relax* or MeSH descriptor Cognitive Therapy explode all trees or MeSH descriptor Behavior Therapy explode all trees or (behavio*r*) NEAR/4 (modif* or therap* or rehab* or change) or MeSH descriptor Stress, Psychological explode all trees or stress NEAR manage* or cognitive* NEAR therap* or MeSH descriptor Meditation explode all trees or meditat*  #24: #7 or #8 or #9 or #10 or #11 or #12 or #13 or #14 or #15 or #16 or #17 or #18 or #19 or #20 or #21 or #22 or #23  #25. random*.ti,ab. or factorial*.ti,ab. or (crossover* or cross over* or cross-over*).ti,ab. or placebo*.ti,ab. or (doubl* adj blind*).ti,ab. or (singl* adj blind*).ti,ab.or assign*.ti,ab. or allocat*.ti,ab. or volunteer*.ti,ab. or CROSSOVER PROCEDURE.sh. or DOUBLE-BLIND PROCEDURE.sh. or RANDOMIZED CONTROLLED TRIAL.sh. or SINGLE BLIND PROCEDURE.sh. or cohort*.ti,ab. or cohort analysis/ or prospective study/ or case*.ti,ab.  #26. Comparative Study/ or exp Evaluation Studies/ or exp Follow Up Studies/ or exp Prospective Studies/ or exp Epidemiologic Studies/ or exp Case Control Studies/ or exp Cohort Studies/ or (cohort adj (study or studies)).mp. or cohort analy$.mp. or (follow up adj (study or studies)).mp. or (observational adj (study or studies)).mp. or retrospective.mp. or prospective$.mp.  #27: #25 or #26  #28. prevention* or protect* or protective or prevent  #29: #6 and #24 and #27 and #28 | 558 |
|  |  |  |
| CENTRAL ClinicalTrials | #1 congnitive decline  #2 nonpharmacological interventions or cognitive training or exercise or dietary | 454 |
|  |  |  |

**Table S1** Diagnosis of MCI or dementia in the included trials

| First author | Authors’ judgement | How to diagnose MCI or dementia |
| --- | --- | --- |
| Linda 2012 | High risk | 1. Progression to dementia: Clinical diagnosis of dementia was made with the DSM-IV criteria. The primary outcome indicator was the rate of “conversion” to clinical dementia using DSM-IV criteria compared between I and C groups after 1 year of intervention. Staging of dementia was evaluated by the CDR, a semistructured interview of 6 dimensions, including memory,orientation, judgment, community affairs, hobbies and habits, and personal care. A CDR of 1 to 3 indicates mild to severe dementia.  2. Cognitive test scores: Differences in the change in cognitive scores from baseline between the I and C groups was considered as cognitive outcome indicator. The Cantonese version of the ADAS-Cog, digit span, delay recall, category verbal fluency tests, trail making, and Mini-Mental State Examination were assessed. |
| Kryscio 2017 | Low risk | Dementia case ascertainment relied on a consensus review of the cognitive screens and medical records for men with suspected dementia who visited their physician for an evaluation or by review of all available information, including a functional assessment screen. Dementia incidence, the primary end point, was determined by 1 of 2 methods. First, if participants failed both the first tier of the screen (MIS score ≤ 5 of 8) and the second tier (T Score ≤ 35 on the CERAD battery; total score ≤35 on the TICS-m), then they were encouraged to obtain a memory workup from their local clinician and share medical records with PREADViSE trial investigators. Medical records were reviewed by a team of 2 to 3 expert neurologists and 2 to 3 expert neuropsychologists to determine consensus diagnoses. Participants who did not obtain the workup were assessed by additional longitudinal measures collected during the study. These included the Ascertain Dementia 8-Item Informant Questionnaire (AD 8) Dementia Screening Interview, self-reported medical history, self-reported medication use, and cognitive scores, including the MIS, CERAD T Score, New York University Paragraph Delayed Recall, and TICS-m. An AD 8 of 1 or greater (at any time during follow-up) as well as a self-reported dementia diagnosis, use of amemory-enhancing prescription drug (eg, donepezil, rivastigmine, galantamine, ormemantine), or cognitive score 1.5 SDs or more below expected performance yielded a dementia diagnosis. The diagnosis date was assigned to the earliest event. |
| Petrelli 2014 | High risk | Definition of MCI:  Subjective cognitive impairment or impairment recognized by the clinician and largely intact activities of daily living according to medical history Neuropsychological test battery, scores of ≤1.5 SD below the population norm mean defined as impaired  Attention: two subtests (numbers and letters) of the Brief Test of Attention.  Memory: DemTect word list (direct and delayed recall) and the delayed recall of the Rey Complex Figure Test.  Executive functions: DemTect working memory and DemTect semantic verbal fluency task(‘supermarket’) and letter verbal fluency task (controlled oral word association – FAS).  Visuo-construction: copy tasks of the Rey Complex Figure Test and pentagon drawing of the MMSE.  Language: MMSE language items. |
| Sink 2015 | Low risk | Participants who scored 88 points or less on the 3MSE were sent for central adjudication by a panel (blinded to treatment assignment) of 8 clinical experts in the diagnosis of late-life cognitive impairment. Each case was assigned to 2 independent adjudicators; disagreements were resolved by the full panel. Based on 2011 criteria from the National Institute on Aging and the Alzheimer’s Association, MCI and dementia were adjudicated. |
| Lapiscina 2013 | Low risk | Medical records of all participants were checked to collect incidence events including mild cognitive impairment (MCI), dementia and depression. This information was sent to The Adjudication Committee. This Committee reviewed the suitability of diagnoses according to the available information. Additionally, participants with a pathological cognitive screening test were re-evaluated by a neurologist to determine the presence or absence of MCI or dementia. Diagnoses of dementia or MCI from the Adjudication Committee were based on assessments recorded in clinical records and usually made by neurologists upon the request of either general practitioners or participants. In addition, we performed a comprehensive cognitive evaluation to identify MCI or dementia. The use of both methods (review of medical record based on referrals by general practitioners and personalised comprehensive neuropsychiatric assessment) are likely to have increased the sensitivity in detecting MCI and dementia. |
| Edwards 2017 | Low risk | Defined dementia as the first occurrence of any of the following:  1. Cognitive and functional impairment defined as follows: a) memory composite score at or below -1.5 SD of the baseline sample mean and reasoning composite, speed composite, or vocabulary score at or below -1.5 SD of the baseline mean (for assessment details see [Jobe JB, Smith DM, Ball KK, Tennstedt SL, Marsiske M, Willis SL,et al. ACTIVE: A cognitive intervention trial to promote independence in older adults. Control Clin Trials 2001;22:453–79]), and b) MDS IADL total score at or below the 10th percentile of the baseline (self-reported).  2. A score of ,22 on the MMSE, with all subsequent MMSE assessments at ,22 or missing [McDowell I, Kristjansson B, Hill GB, Hebert R. Community screening for dementia: The Mini Mental State Exam (MMSE) and modified Mini-Mental State Exam (3MS) compared. J Clin Epidemiol 1997;50:377–83].  3. Self- or proxy-report of diagnosis of dementia or Alzheimer’s disease during the follow-up. |
| Shi 2017 | High risk | According to Diagnostic and Statistical Manual of Mental Disorders of American Psychiatric Association (DSM IV) [Anxiety disorders in the fourth edition of the classification of mental disorders prepared by the American psychiatric association: diagnostic and statistical manual of mental disorders (DMS-IV-ptions book]. Psychiatr Pol 1994; 28: 255-68]. |
| DeKosky 2018 | Low risk | The panel consisted of 2 neurologists with expertise in dementia diagnosis; 2 neuropsychologists experienced in cognitive assessment of dementia; and a psychometrician with extensive experience in training, administration, and scoring of the CDR. Participants classified as reaching dementia end point at this point were then referred for a full neurological evaluation and a magnetic resonance imaging(MRI) scan at the clinical site to confirm that the participant met clinical criteria for dementia and assess for atypical causes of dementia. The MRIs were reviewed according to a standard protocol by 2 boardcertified neuroradiologists, also blinded to treatment assignment, and ratings were given for cortical atrophy; ventricular size; subcortical white matter lesions; and presence, size, and number of brain infarcts. These data and the neuroradiologists’ clinical readings were available to the adjudication panel in its diagnostic decision-making, and the adjudicating neurologists also reviewed the scans themselves as part of their diagnostic process. |


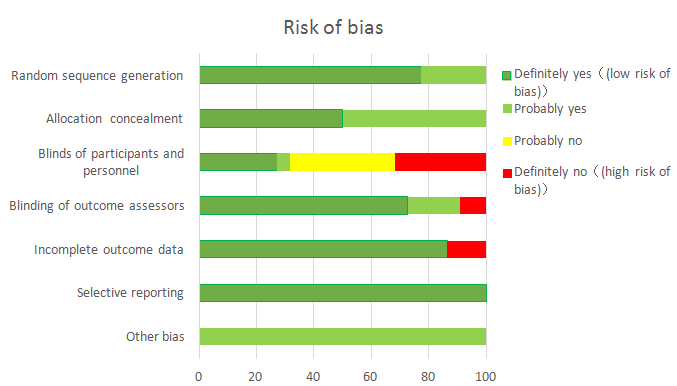


1. **Risk of bias graph**

| First author（years） | Publication year | Random sequence generation | Allocation concealment | Blinds of participants and personnel | Blinding of outcome assessors | Incomplete outcome data | Selective reporting | Other bias |
| --- | --- | --- | --- | --- | --- | --- | --- | --- |
| Linda | 2012 | Definitely yes（(low risk of bias)） | Definitely yes（(low risk of bias)） | Definitely no（(high risk of bias)） | Definitely yes（(low risk of bias)） | Definitely no（(high risk of bias)） | Definitely yes（(low risk of bias)） | Probably yes |
| Kwok | 2012 | Probably yes | Probably yes | Probably yes | Definitely yes（(low risk of bias)） | Definitely yes（(low risk of bias)） | Definitely yes（(low risk of bias)） | Probably yes |
| Vanessa | 2018 | Definitely yes（(low risk of bias)） | Definitely yes（(low risk of bias)） | Definitely yes（(low risk of bias)） | Probably yes | Definitely yes（(low risk of bias)） | Definitely yes（(low risk of bias)） | Probably yes |
| Karin | 2010 | Definitely yes（(low risk of bias)） | Definitely yes（(low risk of bias)） | Definitely yes（(low risk of bias)） | Definitely no（(high risk of bias)） | Definitely yes（(low risk of bias)） | Definitely yes（(low risk of bias)） | Probably yes |
| Daniela | 2014 | Probably yes | Probably yes | Probably yes | Definitely yes（(low risk of bias)） | Definitely yes（(low risk of bias)） | Definitely yes（(low risk of bias)） | Probably yes |
| Lapiscina | 2013 | Definitely yes（(low risk of bias)） | Definitely yes（(low risk of bias)） | Definitely yes（(low risk of bias)） | Definitely yes（(low risk of bias)） | Definitely no（(high risk of bias)） | Definitely yes（(low risk of bias)） | Probably yes |
| Petrelli | 2014 | Definitely yes（(low risk of bias)） | Probably yes | Definitely yes（(low risk of bias)） | Definitely yes（(low risk of bias)） | Definitely no（(high risk of bias)） | Definitely yes（(low risk of bias)） | Probably yes |
| McDougall | 2010 | Definitely yes（(low risk of bias)） | Probably yes | Probably no | Probably yes | Definitely yes（(low risk of bias)） | Definitely yes（(low risk of bias)） | Probably yes |
| Simone | 2006 | Definitely yes（(low risk of bias)） | Probably yes | Probably no | Definitely yes（(low risk of bias)） | Definitely yes（(low risk of bias)） | Definitely yes（(low risk of bias)） | Probably yes |
| Jagadish K | 2018 | Definitely yes（(low risk of bias)） | Definitely yes（(low risk of bias)） | Definitely no（(high risk of bias)） | Definitely yes（(low risk of bias)） | Definitely yes（(low risk of bias)） | Definitely yes（(low risk of bias)） | Probably yes |
| Piedra | 2017 | Probably yes | Probably yes | Probably no | Probably yes | Definitely yes（(low risk of bias)） | Definitely yes（(low risk of bias)） | Probably yes |
| Arnaud | 2015 | Definitely yes（(low risk of bias)） | Definitely yes（(low risk of bias)） | Definitely no（(high risk of bias)） | Definitely no（(high risk of bias)） | Definitely yes（(low risk of bias)） | Definitely yes（(low risk of bias)） | Probably yes |
| Hiroyuki | 2018 | Definitely yes（(low risk of bias)） | Definitely yes（(low risk of bias)） | Definitely no（(high risk of bias)） | Definitely yes（(low risk of bias)） | Definitely yes（(low risk of bias)） | Definitely yes（(low risk of bias)） | Probably yes |
| Cinta Valls | 2015 | Definitely yes（(low risk of bias)） | Probably yes | Probably no | Definitely yes（(low risk of bias)） | Definitely yes（(low risk of bias)） | Definitely yes（(low risk of bias)） | Probably yes |
| Antonio | 2009 | Probably yes | Probably yes | Probably no | Definitely yes（(low risk of bias)） | Definitely yes（(low risk of bias)） | Definitely yes（(low risk of bias)） | Probably yes |
| Shi | 2017 | Definitely yes（(low risk of bias)） | Probably yes | Probably no | Probably yes | Definitely yes（(low risk of bias)） | Probably yes | Probably yes |
| Kryscio | 2017 | Definitely yes（(low risk of bias)） | Definitely yes（(low risk of bias)） | Definitely yes（(low risk of bias)） | Definitely yes（(low risk of bias)） | Definitely yes（(low risk of bias)） | Definitely yes（(low risk of bias)） | Definitely yes（(low risk of bias)） |
| Sink | 2015 | Definitely yes（(low risk of bias)） | Definitely yes（(low risk of bias)） | Definitely no（(high risk of bias)） | Definitely yes（(low risk of bias)） | Definitely yes（(low risk of bias)） | Definitely yes（(low risk of bias)） | Definitely yes（(low risk of bias)） |
| Edwards | 2017 | Definitely yes（(low risk of bias)） | Probably yes | Definitely no（(high risk of bias)） | Definitely yes（(low risk of bias)） | Definitely yes（(low risk of bias)） | Definitely yes（(low risk of bias)） | Probably yes |
|  |  |  |  |  |  |  |  |  |
|  |  |  |  |  |  |  |  |  |
|  |  |  |  |  |  |  |  |  |
|  |  |  |  |  |  |  |  |  |
|  |  |  |  |  |  |  |  |  |
|  |  |  |  |  |  |  |  |  |
|  |  |  |  |  |  |  |  |  |
|  |  |  |  |  |  |  |  |  |
| DeKosky | 2018 | Definitely yes（(low risk of bias)） | Definitely yes（(low risk of bias)） | Definitely yes（(low risk of bias)） | Definitely yes（(low risk of bias)） | Definitely yes（(low risk of bias)） | Definitely yes（(low risk of bias)） | Definitely yes（(low risk of bias)） |
|  |  |  |  |  |  |  |  |  |
|  |  |  |  |  |  |  |  |  |
|  |  |  |  |  |  |  |  |  |
|  |  |  |  |  |  |  |  |  |
|  |  |  |  |  |  |  |  |  |
|  |  |  |  |  |  |  |  |  |
|  |  |  |  |  |  |  |  |  |
|  |  |  |  |  |  |  |  |  |
|  |  |  |  |  |  |  |  |  |
|  |  |  |  |  |  |  |  |  |
| Lautenschlager | 2008 | Definitely yes（(low risk of bias)） | Definitely yes（(low risk of bias)） | Definitely yes（(low risk of bias)） | Definitely yes（(low risk of bias)） | Definitely yes（(low risk of bias)） | Definitely yes（(low risk of bias)） | Definitely yes（(low risk of bias)） |
|  |  |  |  |  |  |  |  |  |
|  |  |  |  |  |  |  |  |  |
| Olivia | 2016 | Probably yes | Probably yes | Probably no | Definitely yes（(low risk of bias)） | Definitely yes（(low risk of bias)） | Probably yes | Definitely no（(high risk of bias)） |

**(B) Risk of bias summary**

**Fig. S1** Assessment of risk of bias: (A) Risk of bias graph and (B) Risk of bias summary


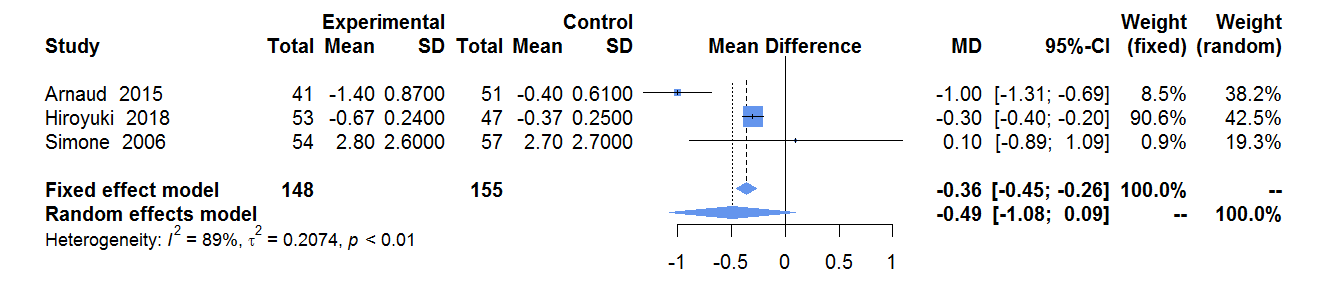


**Fig. S2** Forest plot of GDS

**Table S2 Summary of Strength of Evidence for Outcomes**

| **Certainty assessment** | | | | | | | **№ of patients** | | **Effect** | | **Certainty** | **Importance** |
| --- | --- | --- | --- | --- | --- | --- | --- | --- | --- | --- | --- | --- |
| **№ of studies** | **Study design** | **Risk of bias** | **Inconsistency** | **Indirectness** | **Imprecision** | **Other considerations** | **Nonpharmacologic** | **Control** | **Relative (95% CI)** | **Absolute (95% CI)** |
| **the incidence of MCI or dementia** | | | | | | | | | | | | |
| 8 | randomised trials | not serious | serious ^a^ | not serious | not serious | none | 551/4977 (11.1%) | 589/4956 (11.9%) | **RR 0.73** (0.55 to 0.96) | **32 fewer per 1,000** (from 53 fewer to 5 fewer) | ⨁⨁⨁◯ MODERATE | CRITICAL |
| **ADAS-Cog** | | | | | | | | | | | | |
| 2 | randomised trials | serious ^b^ | very serious ^a,c^ | not serious | very serious ^d^ | none | 82 | 82 | - | MD **0.69 lower** (1.52 lower to 0.14 higher) | ⨁◯◯◯ VERY LOW | CRITICAL |
| **ADL** | | | | | | | | | | | | |
| 2 | randomised trials | serious ^b^ | not serious | not serious | serious ^e^ | none | 122 | 134 | - | MD **0.73 higher** (0.65 higher to 0.8 higher) | ⨁⨁◯◯ LOW | IMPORTANT |
| **MMSE** | | | | | | | | | | | | |
| 9 | randomised trials | not serious | serious ^a,c^ | not serious | serious ^c^ | none | 1168 | 1140 | - | MD **0.04 higher** (0.04 lower to 0.12 higher) | ⨁⨁◯◯ LOW | IMPORTANT |
| **MMSE(ΔE vs. ΔC)** | | | | | | | | | | | | |
| 6 | randomised trials | not serious | serious ^a^ | not serious | not serious | none | 763 | 743 | - | MD **0.5 higher** (0.46 higher to 0.53 higher) | ⨁⨁⨁◯ MODERATE | IMPORTANT |
| **GDS** | | | | | | | | | | | | |
| 3 | randomised trials | serious ^b^ | very serious ^a,c^ | not serious | very serious ^d^ | none | 148 | 155 | - | MD **0.36 lower** (0.45 lower to 0.26 lower) | ⨁◯◯◯ VERY LOW | IMPORTANT |

**CI:** Confidence interval; **RR:** Relative risk; **MD:** Mean difference

#### Explanations

a. Downgraded one level for heterogeneity : the statistical test for heterogeneity showed that large variation (I² >50%) existed in point estimates due to among-study differences.

b. Downgraded one level for risk of bias: most of the included RCTs had unclear risk of concealment of allocation

c. 95% Confidence intervals(CIs) around the pooled included no effect and appreciable benefit

d. Downgraded two levels for imprecision: the sample size was less than 300, the number of events not high, 95% Confidence intervals(CIs) show overlap and 95% Confidence intervals(CIs) crossed the line of no effect and appreciable benefit

e. Downgraded one level: total sample size is lower than 300

**
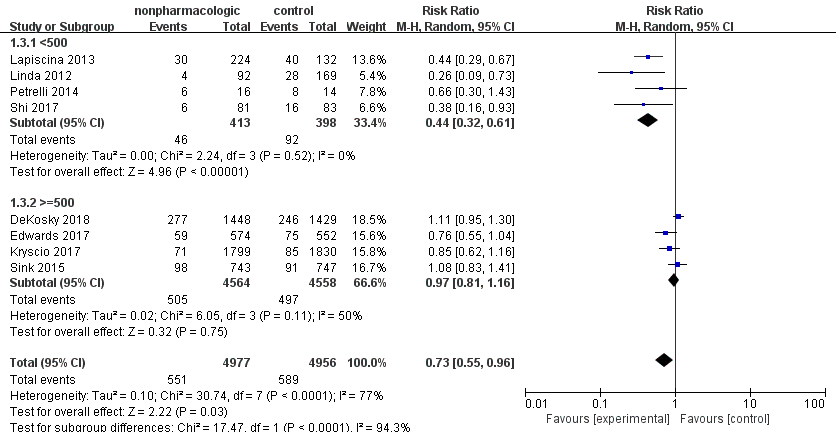
**

1. Forest plot of subgroups by the number of sample size(< 500 and ≥ 500)


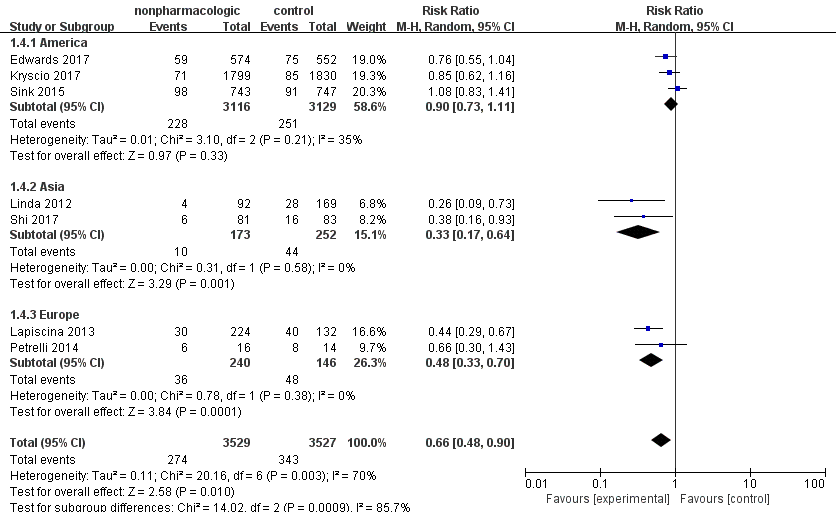


1. Forest plot of subgroups by the area of RCTs(America, Asia and Europe)

**
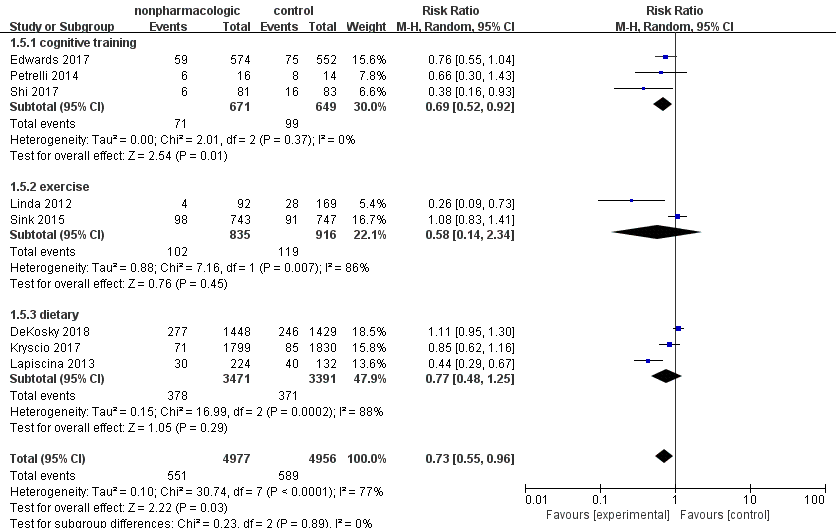
**

1. Forest plot of subgroups by the type of nonpharmacological interventions(cognitive training,exercise and dietary)

**
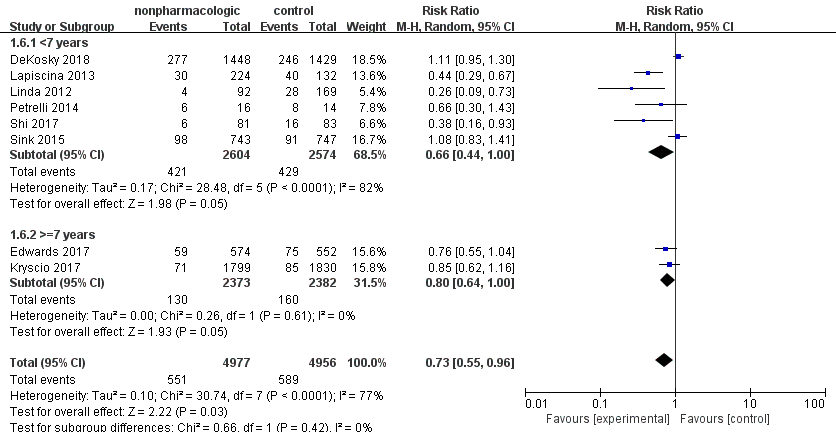
**

1. Forest plot of subgroups by the duration of follow-up(< 7 years and ≥ 7 years)

**Fig. S3** Forest plot of subgroups

1. Forest plot of subgroups by the number of sample size(< 500 and ≥ 500)
2. Forest plot of subgroups by the area of RCTs(America, Asia and Europe)
3. Forest plot of subgroups by the type of nonpharmacological interventions(cognitive training,exercise and dietary)
4. Forest plot of subgroups by the duration of follow-up(< 7 years and ≥ 7 years)


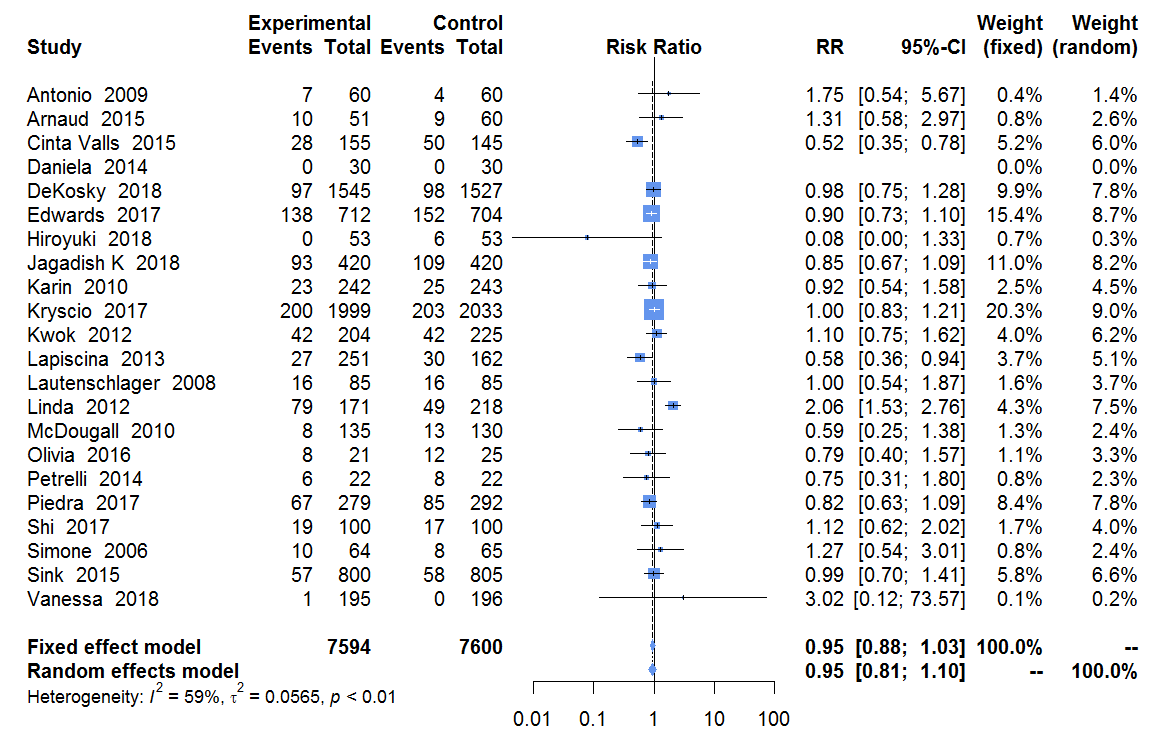


**Fig. S4** Forest plot of prevention acceptability


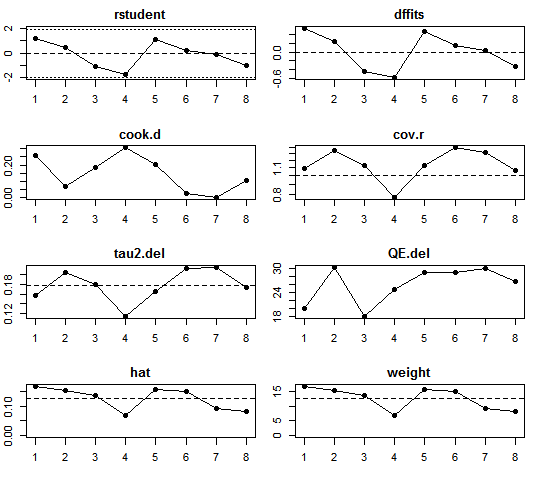


**(A) Influence Analyses**


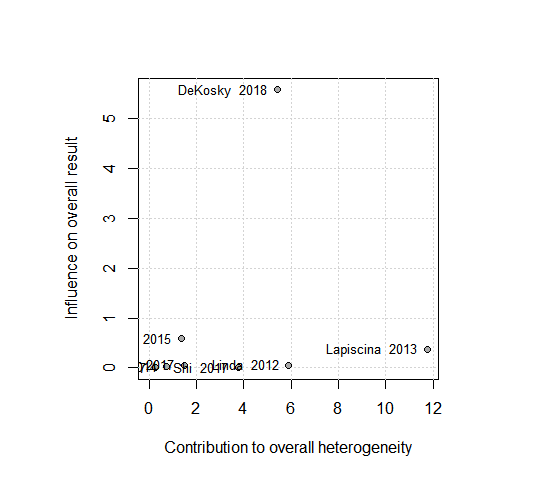


1. **Baujat Plot**


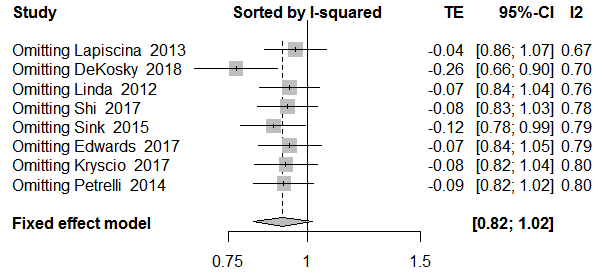


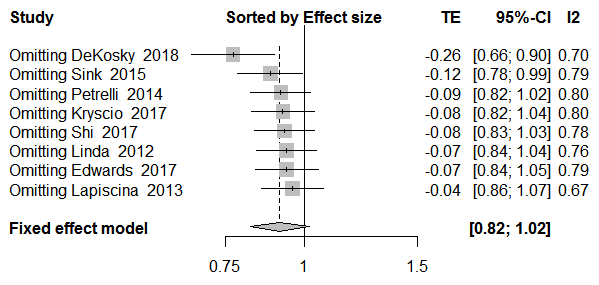


**(C) Leave-One-Out-Analyses**

**Fig. S5 Outlier and influence analysis**

**(A) Influence Analyses**

X-axis represents 8 trials that reported the incidence of MCI or dementia

Y-axis

**rstudent:** The studentized deleted residual for a particular study formalizes a proper outlier test under a mean shift outlier model.

**dffits:** The DIFFITS value of a study indicates in standard deviations how much the predicted pooled effect changes after excluding this study.

**cook.d:** The Cook’s distance resembles the Mahalanobis distance you may know from outlier detection in conventional multivariate statistics. It is the distance between the value once the study is included compared to when it is excluded.

**cov.r:** The covariance ratio is the determinant of the variance-covariance matrix of the parameter estimates when the study is removed, divided by the determinant of the variance-covariance matrix of the parameter estimates when the full dataset is considered. Importantly, values of cov.r < 1 indicate that removing the study will lead to a more precise effect size estimation (i.e., less heterogeneity).

**tau2.del:** It is one type of heterogeneity measures.

**QE:** QE is the statistic of the homogeneity test

**hat:** hat values.

1. **Baujat Plot**

**(C) Leave-One-Out-Analyses**


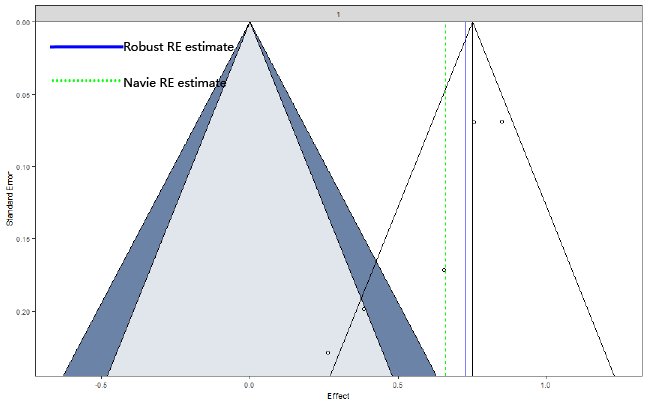


**(A) Contour-enhanced funnel plot of observed nonpharmacological interventions effects from included randomized clinical trials that reported the incidence of MCI or dementia**


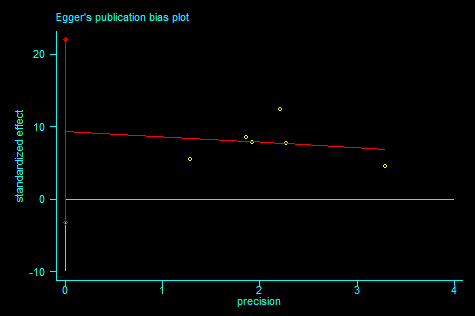


**(B) Egger’s publication bias plot**

**Figure S6. Funnel plot and Egger test**

**(A) Contour-enhanced funnel plot of observed nonpharmacological interventions effects from included randomized clinical trials that reported the incidence of MCI or dementia**

**(B) Egger’s publication bias plot**
