# Supplementary material for: Do nonpharmacological interventions prevent cognitive decline? a systematic review and meta-analysis
Source: Transl Psychiatry. 2020 Jan 21;10:19. doi: 10.1038/s41398-020-0690-4 (PMC7026127; doi:10.1038/s41398-020-0690-4)
Supplement: Supplementary file 2 — Supplement S1 [file 41398_2020_690_MOESM2_ESM.doc]

**Supplement S1** Search strategy for each database

**Table S1**  Diagnosis of MCI or dementia in the included trials

**Fig. S1** Assessment of risk of bias

**Fig. S2** Forest plot of GDS

**Table S2** Summary of strength of evidence for outcomes

**Fig. S3** Forest plot of subgroups

**Fig. S4** Forest plot of prevention acceptability

**Fig. S5** Outlier and influence analysis

**Fig. S6** Funnel plot and Egger test

**Supplement S1.** Search strategy for each database

The search was originally carried out on March 31, 2019.

Databases: Medline, EMBASE, Cochrane library, CENTRAL, ClinicalTrials database

| Database | Search | Included Returns |
| --- | --- | --- |
| EMBASE/  MEDLINE | #1. cognitive decline.mp. or Cognitive Dysfunction/  #2. cognitive impairment.mp. or Cognitive Dysfunction/  #3. Cognitive Dissonance/  #4. (Cognitive Impairment or Cognitive Impairments or Neurocognitive Disorder or Decline, #Cognitive or Mental Deteriorations or Mental Deterioration or Declines, Cognitive or #Cognitive Declines or Cognitive Decline or Deterioration, Mental or Deteriorations, Mental or Neurocognitive Disorders or Dissonance, Cognitive or Dissonances, Cognitive or Cognitive Dissonances or Cognitive Aging).mp.  #5: #1 or #2 or #3 or #4  #6. exp Exercise/ OR exp Exercise Therapy/ OR exp "Physical Education and Training"/ OR Physical Fitness/ OR Physical Exertion/ OR exp Walking/ OR Running/ or Jogging/ OR Swimming/ OR (cycling or bicycling).tw. OR (exercise$ or exercising).tw. OR (physical adj3 (education or training)).tw. OR exp physical activity/ OR exp sport/ OR exp Combined Modality Therapy/ OR Exercise/ OR Physical Exertion/ OR exp Physical Therapy Modalities/ OR (alexander adj (technique or method)).tw. OR Biofeedback, Psychology/  #7. yog*.af. OR Meditation OR meditat*.af. OR Relaxation OR relax*.af. OR yoga* OR meditat* OR relaxation* OR Relaxation Therapy/  #8. hatha OR ashtanga OR bikram OR iyengar OR kripalu OR kundalini OR sivananda OR vinyasa OR raja OR radja OR bhakti OR jnana OR kriya OR karma OR yama OR niyama OR asana OR pranayama OR pratyahara OR dharana OR dhyana OR samadhi OR bandha OR mudra  #9. PLYOMETRIC EXERCISE/ or COOL-DOWN EXERCISE/ or EXERCISE/ or EXERCISE MOVEMENT TECHNIQUES/ or EXERCISE THERAPY/ or WARM-UP EXERCISE/  #10. Psychology,Social.mp. or Psychology, Social/  #11. PSYCHOTHERAPY, RATIONAL-EMOTIVE/ or PSYCHOTHERAPY/ or PSYCHOTHERAPY, MULTIPLE/ or "IMAGERY (PSYCHOTHERAPY)"/ or PSYCHOTHERAPY, GROUP/ or Psychotherapy.mp. or PSYCHOTHERAPY, BRIEF/ or PSYCHOTHERAPY, PSYCHODYNAMIC/  #12. PSYCHOTHERAP$2.tw. OR RELAX$6.tw. OR (PSYCHOLOG$5 adj4 INTERVENT$5).tw. OR Relaxation Training/ OR exp Counseling/ OR (COUNSELLING or COUNSELING).tw. OR ((BEHAVIOR$4 or BEHAVIOUR$4) adj4 (MODIFY or MODIFICAT$4 or THERAPY$2 or CHANGE)).mp. OR Stress Management/ OR ((BEHAVIOR$4 or BEHAVIOUR$4) adj4 (MODIFY or MODIFICAT$4 or THERAPY$2 or CHANGE)).tw. OR (STRESS adj3 MANAGEMENT).tw. OR exp Meditation/ OR MEDITAT$5.tw. #13. (MANAGE$5 adj2 (ANXIETY or DEPRES$5)).tw. OR CBT.tw. OR HYPNOTHERAP$2.tw. OR (GOAL$2 adj3 SETTING).tw. OR (MOTIVAT$5 adj4 INTERVENT$6).tw. OR Psychosocial Care/ OR Psychosocial Rehabilitation/ OR PSYCHOSOCIAL.tw. OR Autogenic Training/ OR AUTOGENIC.tw.  #14. COGNITIVE THERAPY/ OR BEHAVIOR THERAPY/  #15. (cogniti$ adj3 (behavio$ or intervention$ or psychotherap$ or technique$ or therap$ or treat$)).ti,ab.  #16. (behavio$ adj3 (intervention$ or psychotherapy$ or technique$ or therap$ or treat$)).ti,ab.  #17. Cognitive Therapy.mp. or Cognitive Therapy/ OR (cognit* adj2 stimulation).mp. OR (cognit* adj2 rehabilitation).mp or (cognit* adj2 training).mp. or (cognit* adj2 retrain*).mp. OR cognitive support.mp. OR memory function.mp. OR (memory adj2 rehabilitation).mp. OR (memory adj2 therap*).mp.or memory aid.mp. OR memory group.mp. OR memory training.mp. OR memory retraining.mp. OR memory support.mp OR memory stimulation.mp. OR memory management.mp.  #18.Cognitive Therapy.mp. or Cognitive Therapy/ or (cognit* adj2 stimulation).mp.or  (cognit* adj2 rehabilitation).mp or (cognit* adj2 training).mp. or (cognit* adj2 retrain*).mp. or cognitive support.mp. or memory function.mp. or (memory adj2 rehabilitation).mp. or (memory adj2 therap*).mp.or memory aid.mp. or memory group.mp. or memory training.mp. or memory retraining.mp. or memory support.mp or memory stimulation.mp. or memory management.mp.  #19.exp Electric Stimulation Therapy/ OR exp Rehabilitation/ OR Hydrotherapy/ OR postur* correction.mp. OR Feldenkrais.mp.  #20. Complementary Therapies.mp. or Complementary Therapies/  #21. (Therapies, Complementary or Therapy, Complementary or Complementary Medicine or Medicine, Complementary or Alternative Medicine or Medicine, Alternative or Alternative Therapies or Therapies, Alternative or Therapy, Alternative).mp.  #22. Taichi or Taiji or Ba duan jin  #23. (art therapy or Reminiscence therapy or Validation therapy or Simulated presence therapy or Light therapy).mp.  #24. (Animal therapy or Aromatherapy or Snoezelen room Exercise or Cognitive training and rehabilitation or group exercise or walking programs or cognitive stimulation treatment).mp.  #25. (progressive muscle relaxation or cognitive training or Physiotherapy or acupuncture or Homeopathy or Hynosis or Massage or Naturopathy or Osteopathy or relaxation).mp.  #26: #6 or #7 or #8 or #9 or #10 or #11 or #12 or #13 or #14 or #15 or #16 or #17 or #18 or #19 or #20 or #21 or #22 or #23 or #24 or #25  #27. random*.ti,ab. or factorial*.ti,ab. or (crossover* or cross over* or cross-over*).ti,ab. or placebo*.ti,ab. or (doubl* adj blind*).ti,ab. or (singl* adj blind*).ti,ab.or assign*.ti,ab. or allocat*.ti,ab. or volunteer*.ti,ab. or CROSSOVER PROCEDURE.sh. or DOUBLE-BLIND PROCEDURE.sh. or RANDOMIZED CONTROLLED TRIAL.sh. or SINGLE BLIND PROCEDURE.sh. or cohort*.ti,ab. or cohort analysis/ or prospective study/ or case*.ti,ab.  #28. Comparative Study/ or exp Evaluation Studies/ or exp Follow Up Studies/ or exp Prospective Studies/ or exp Epidemiologic Studies/ or exp Case Control Studies/ or exp Cohort Studies/ or (cohort adj (study or studies)).mp. or cohort analy$.mp. or (follow up adj (study or studies)).mp. or (observational adj (study or studies)).mp. or retrospective.mp. or prospective$.mp.  #29: #27 or #28  #30. prevention* or protect* or protective or prevent  #31: #5 and 26 and #29 and #30 | EMBASE: 5232  MEDLINE: 1376 |
| Cochrane | #1. MeSH descriptor: [cognitive decline] explode all trees  #2. MeSH descriptor: [cognitive dysfunction] explode all trees  #3. MeSH descriptor: [cognitive impairment] explode all trees  #4. MeSH descriptor: [Cognitive Dissonance] explode all trees  #5.（ Cognitive Impairment OR Cognitive Impairments OR Neurocognitive Disorder OR Decline, Cognitive OR Mental Deteriorations OR Mental Deterioration OR Declines, Cognitive OR Cognitive Declines OR Cognitive Decline OR Deterioration, Mental OR Deteriorations, Mental OR Neurocognitive Disorders OR Dissonance, Cognitive OR Dissonances, Cognitive OR Cognitive Dissonances OR Cognitive Aging ）:ti,ab,kw (Word variations have been searched)  #6: #1 or #2 or #3 or #4 or #5  #7. MeSH descriptor: [Psychology,Social.] explode all trees  #8. MeSH descriptor: [Psychotherapy] explode all trees  #9.(incentive*OR voucher OR psychotherap* OR psychosocial* OR behaviour therapy OR behavior therapy OR reinforcement OR motivation* OR contingent* OR advice OR biofeedback OR community OR stimulation OR education* OR brief intervention OR early intervention OR minimal intervention OR counseling OR counsel* OR cognitive therapy OR family therapy OR social skill OR stress management training OR supportive expressive therapy OR neurobehavioral* OR coping skill* OR "self-control training"):ti,ab,kw (Word variations have been searched)  #10. MeSH descriptor: [Complementary Therapies] explode all trees  #11. (Therapies, Complementary or Therapy, Complementary or Complementary Medicine or Medicine, Complementary or Alternative Medicine or Medicine, Alternative or Alternative Therapies or Therapies, Alternative or Therapy, Alternative):ti,ab,kw (Word variations have been searched)  #12. MeSH descriptor: [exercise] explode all trees  #13. (Yoga OR art therapy OR Reminiscence therapy OR Validation therapy OR Simulated presence therapy OR Light therapy OR Animal therapy OR Aromatherapy OR Snoezelen room Exercise OR Cognitive training and rehabilitation OR group exercise OR walking programs OR cognitive stimulation treatment OR mindfulness-based Alzheimer’s stimulation OR progressive muscle relaxation OR cognitive training OR Physiotherapy) :ti,ab,kw (Word variations have been searched)  #14. Synonyms: Exercise, Acute; Exercises, Acute; Acute Exercises; Acute Exercise; Physical Activities; Activities, Physical; Exercises; Activity, Physical; Physical Activity; Exercises, Physical; Physical Exercise; Exercise, Physical; Physical Exercises; Exercise, Aerobic; Aerobic Exercise; Exercises, Aerobic; Aerobic Exercises; Training, Exercise; Exercise Trainings; Trainings, Exercise; Exercise Training; Isometric Exercise; Exercise, Isometric; Exercises, Isometric; Isometric Exercises  #15. MeSH DESCRIPTOR Behavior Therapy Explode All  #16. MeSH DESCRIPTOR Psychotherapy  #17. CBT:TI,AB,KW  #18. cognitiv* NEAR3 (behav* or treatment* or technique* or therap* or intervention* or restructur* or reappraisal*)  #19. behav* NEAR3 (treatment* OR therap* or intervention* OR activat* or technique* or modif* or change*)  #20. coping* NEAR3 (skill* or strateg*)  #21. psychotherap* or psychological*  #22. MeSH descriptor Psychotherapy explode all trees #23. psychotherap* or psycholog* NEAR intervent* or relax* or MeSH descriptor Cognitive Therapy explode all trees or MeSH descriptor Behavior Therapy explode all trees or (behavio*r*) NEAR/4 (modif* or therap* or rehab* or change) or MeSH descriptor Stress, Psychological explode all trees or stress NEAR manage* or cognitive* NEAR therap* or MeSH descriptor Meditation explode all trees or meditat*  #24: #7 or #8 or #9 or #10 or #11 or #12 or #13 or #14 or #15 or #16 or #17 or #18 or #19 or #20 or #21 or #22 or #23  #25. random*.ti,ab. or factorial*.ti,ab. or (crossover* or cross over* or cross-over*).ti,ab. or placebo*.ti,ab. or (doubl* adj blind*).ti,ab. or (singl* adj blind*).ti,ab.or assign*.ti,ab. or allocat*.ti,ab. or volunteer*.ti,ab. or CROSSOVER PROCEDURE.sh. or DOUBLE-BLIND PROCEDURE.sh. or RANDOMIZED CONTROLLED TRIAL.sh. or SINGLE BLIND PROCEDURE.sh. or cohort*.ti,ab. or cohort analysis/ or prospective study/ or case*.ti,ab.  #26. Comparative Study/ or exp Evaluation Studies/ or exp Follow Up Studies/ or exp Prospective Studies/ or exp Epidemiologic Studies/ or exp Case Control Studies/ or exp Cohort Studies/ or (cohort adj (study or studies)).mp. or cohort analy$.mp. or (follow up adj (study or studies)).mp. or (observational adj (study or studies)).mp. or retrospective.mp. or prospective$.mp.  #27: #25 or #26  #28. prevention* or protect* or protective or prevent  #29: #6 and #24 and #27 and #28 | 558 |
|  |  |  |
| CENTRAL ClinicalTrials | #1 congnitive decline  #2 nonpharmacological interventions or cognitive training or exercise or dietary | 454 |
|  |  |  |
