# Supplementary material for: Do nonpharmacological interventions prevent cognitive decline? a systematic review and meta-analysis
Source: Transl Psychiatry. 2020 Jan 21;10:19. doi: 10.1038/s41398-020-0690-4 (PMC7026127; doi:10.1038/s41398-020-0690-4)
Supplement: Supplementary file 3 — Fig. S1 [file 41398_2020_690_MOESM3_ESM.doc]

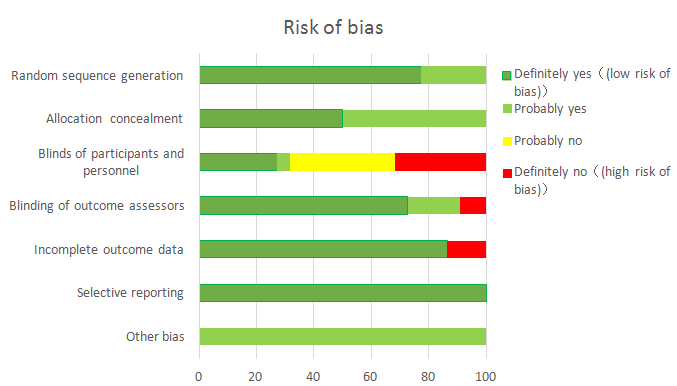


1. **Risk of bias graph**

| First author（years） | Publication year | Random sequence generation | Allocation concealment | Blinds of participants and personnel | Blinding of outcome assessors | Incomplete outcome data | Selective reporting | Other bias |
| --- | --- | --- | --- | --- | --- | --- | --- | --- |
| Linda | 2012 | Definitely yes（(low risk of bias)） | Definitely yes（(low risk of bias)） | Definitely no（(high risk of bias)） | Definitely yes（(low risk of bias)） | Definitely no（(high risk of bias)） | Definitely yes（(low risk of bias)） | Probably yes |
| Kwok | 2012 | Probably yes | Probably yes | Probably yes | Definitely yes（(low risk of bias)） | Definitely yes（(low risk of bias)） | Definitely yes（(low risk of bias)） | Probably yes |
| Vanessa | 2018 | Definitely yes（(low risk of bias)） | Definitely yes（(low risk of bias)） | Definitely yes（(low risk of bias)） | Probably yes | Definitely yes（(low risk of bias)） | Definitely yes（(low risk of bias)） | Probably yes |
| Karin | 2010 | Definitely yes（(low risk of bias)） | Definitely yes（(low risk of bias)） | Definitely yes（(low risk of bias)） | Definitely no（(high risk of bias)） | Definitely yes（(low risk of bias)） | Definitely yes（(low risk of bias)） | Probably yes |
| Daniela | 2014 | Probably yes | Probably yes | Probably yes | Definitely yes（(low risk of bias)） | Definitely yes（(low risk of bias)） | Definitely yes（(low risk of bias)） | Probably yes |
| Lapiscina | 2013 | Definitely yes（(low risk of bias)） | Definitely yes（(low risk of bias)） | Definitely yes（(low risk of bias)） | Definitely yes（(low risk of bias)） | Definitely no（(high risk of bias)） | Definitely yes（(low risk of bias)） | Probably yes |
| Petrelli | 2014 | Definitely yes（(low risk of bias)） | Probably yes | Definitely yes（(low risk of bias)） | Definitely yes（(low risk of bias)） | Definitely no（(high risk of bias)） | Definitely yes（(low risk of bias)） | Probably yes |
| McDougall | 2010 | Definitely yes（(low risk of bias)） | Probably yes | Probably no | Probably yes | Definitely yes（(low risk of bias)） | Definitely yes（(low risk of bias)） | Probably yes |
| Simone | 2006 | Definitely yes（(low risk of bias)） | Probably yes | Probably no | Definitely yes（(low risk of bias)） | Definitely yes（(low risk of bias)） | Definitely yes（(low risk of bias)） | Probably yes |
| Jagadish K | 2018 | Definitely yes（(low risk of bias)） | Definitely yes（(low risk of bias)） | Definitely no（(high risk of bias)） | Definitely yes（(low risk of bias)） | Definitely yes（(low risk of bias)） | Definitely yes（(low risk of bias)） | Probably yes |
| Piedra | 2017 | Probably yes | Probably yes | Probably no | Probably yes | Definitely yes（(low risk of bias)） | Definitely yes（(low risk of bias)） | Probably yes |
| Arnaud | 2015 | Definitely yes（(low risk of bias)） | Definitely yes（(low risk of bias)） | Definitely no（(high risk of bias)） | Definitely no（(high risk of bias)） | Definitely yes（(low risk of bias)） | Definitely yes（(low risk of bias)） | Probably yes |
| Hiroyuki | 2018 | Definitely yes（(low risk of bias)） | Definitely yes（(low risk of bias)） | Definitely no（(high risk of bias)） | Definitely yes（(low risk of bias)） | Definitely yes（(low risk of bias)） | Definitely yes（(low risk of bias)） | Probably yes |
| Cinta Valls | 2015 | Definitely yes（(low risk of bias)） | Probably yes | Probably no | Definitely yes（(low risk of bias)） | Definitely yes（(low risk of bias)） | Definitely yes（(low risk of bias)） | Probably yes |
| Antonio | 2009 | Probably yes | Probably yes | Probably no | Definitely yes（(low risk of bias)） | Definitely yes（(low risk of bias)） | Definitely yes（(low risk of bias)） | Probably yes |
| Shi | 2017 | Definitely yes（(low risk of bias)） | Probably yes | Probably no | Probably yes | Definitely yes（(low risk of bias)） | Probably yes | Probably yes |
| Kryscio | 2017 | Definitely yes（(low risk of bias)） | Definitely yes（(low risk of bias)） | Definitely yes（(low risk of bias)） | Definitely yes（(low risk of bias)） | Definitely yes（(low risk of bias)） | Definitely yes（(low risk of bias)） | Definitely yes（(low risk of bias)） |
| Sink | 2015 | Definitely yes（(low risk of bias)） | Definitely yes（(low risk of bias)） | Definitely no（(high risk of bias)） | Definitely yes（(low risk of bias)） | Definitely yes（(low risk of bias)） | Definitely yes（(low risk of bias)） | Definitely yes（(low risk of bias)） |
| Edwards | 2017 | Definitely yes（(low risk of bias)） | Probably yes | Definitely no（(high risk of bias)） | Definitely yes（(low risk of bias)） | Definitely yes（(low risk of bias)） | Definitely yes（(low risk of bias)） | Probably yes |
|  |  |  |  |  |  |  |  |  |
|  |  |  |  |  |  |  |  |  |
|  |  |  |  |  |  |  |  |  |
|  |  |  |  |  |  |  |  |  |
|  |  |  |  |  |  |  |  |  |
|  |  |  |  |  |  |  |  |  |
|  |  |  |  |  |  |  |  |  |
|  |  |  |  |  |  |  |  |  |
| DeKosky | 2018 | Definitely yes（(low risk of bias)） | Definitely yes（(low risk of bias)） | Definitely yes（(low risk of bias)） | Definitely yes（(low risk of bias)） | Definitely yes（(low risk of bias)） | Definitely yes（(low risk of bias)） | Definitely yes（(low risk of bias)） |
|  |  |  |  |  |  |  |  |  |
|  |  |  |  |  |  |  |  |  |
|  |  |  |  |  |  |  |  |  |
|  |  |  |  |  |  |  |  |  |
|  |  |  |  |  |  |  |  |  |
|  |  |  |  |  |  |  |  |  |
|  |  |  |  |  |  |  |  |  |
|  |  |  |  |  |  |  |  |  |
|  |  |  |  |  |  |  |  |  |
|  |  |  |  |  |  |  |  |  |
| Lautenschlager | 2008 | Definitely yes（(low risk of bias)） | Definitely yes（(low risk of bias)） | Definitely yes（(low risk of bias)） | Definitely yes（(low risk of bias)） | Definitely yes（(low risk of bias)） | Definitely yes（(low risk of bias)） | Definitely yes（(low risk of bias)） |
|  |  |  |  |  |  |  |  |  |
|  |  |  |  |  |  |  |  |  |
| Olivia | 2016 | Probably yes | Probably yes | Probably no | Definitely yes（(low risk of bias)） | Definitely yes（(low risk of bias)） | Probably yes | Definitely no（(high risk of bias)） |

**(B) Risk of bias summary**

**Fig. S1** Assessment of risk of bias: (A) Risk of bias graph and (B) Risk of bias summary
