# Supplementary figures and images for: Do nonpharmacological interventions prevent cognitive decline? a systematic review and meta-analysis
Source: Transl Psychiatry. 2020 Jan 21;10:19. doi: 10.1038/s41398-020-0690-4 (PMC7026127; doi:10.1038/s41398-020-0690-4)

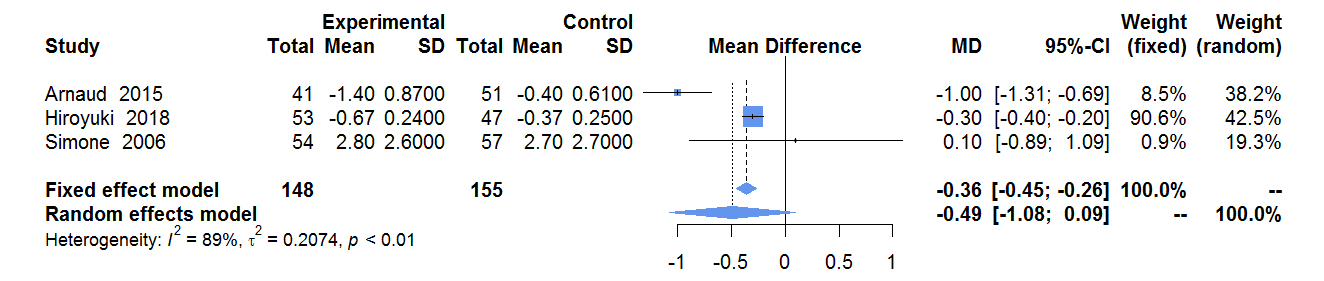


**Fig. S2** Forest plot of GDS

Supplement: Supplementary file 4 — Fig. S2 [file 41398_2020_690_MOESM4_ESM.doc]

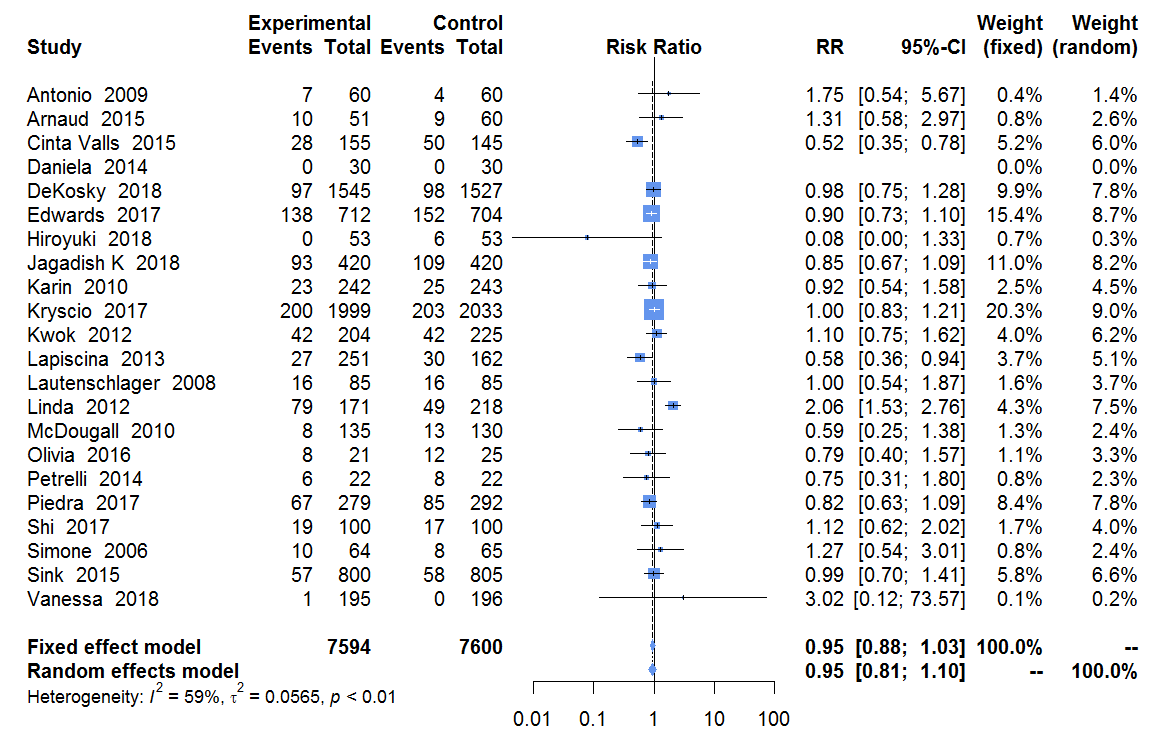


**Fig. S4** Forest plot of prevention acceptability

Supplement: Supplementary file 6 — Fig. S4 [file 41398_2020_690_MOESM6_ESM.doc]
