# Supplementary material for: Do nonpharmacological interventions prevent cognitive decline? a systematic review and meta-analysis
Source: Transl Psychiatry. 2020 Jan 21;10:19. doi: 10.1038/s41398-020-0690-4 (PMC7026127; doi:10.1038/s41398-020-0690-4)
Supplement: Supplementary file 5 — Fig. S3 [file 41398_2020_690_MOESM5_ESM.doc]

**
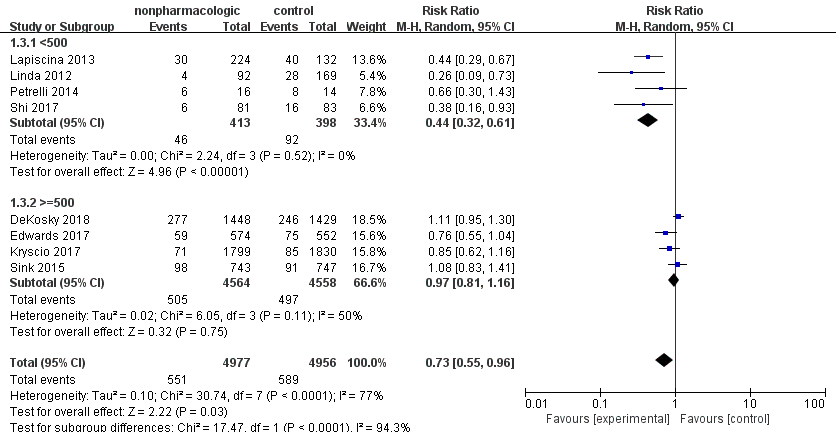
**

1. Forest plot of subgroups by the number of sample size(< 500 and ≥ 500)


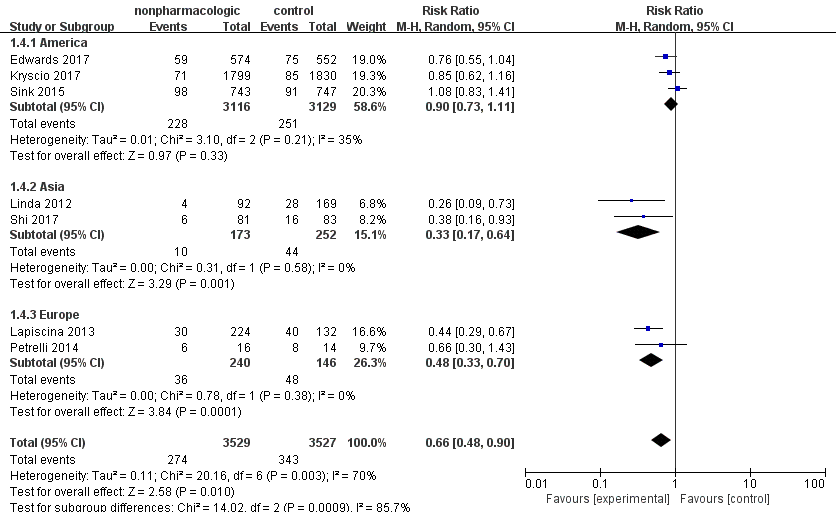


1. Forest plot of subgroups by the area of RCTs(America, Asia and Europe)

**
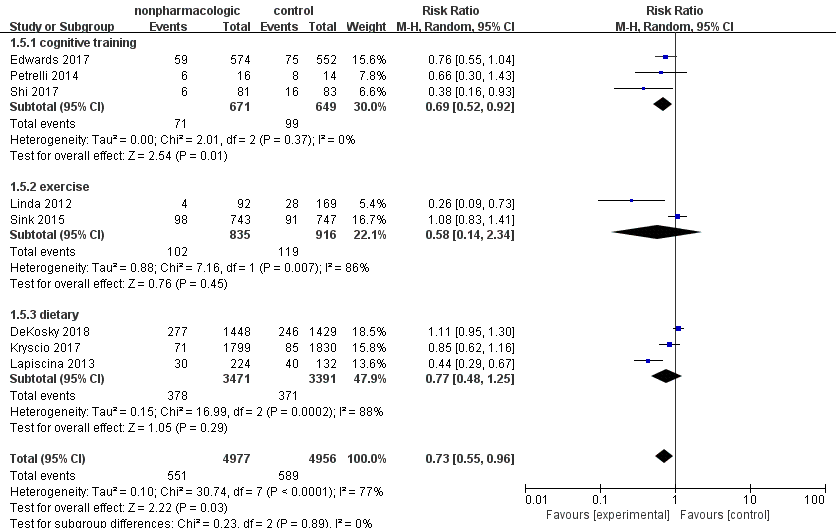
**

1. Forest plot of subgroups by the type of nonpharmacological interventions(cognitive training,exercise and dietary)

**
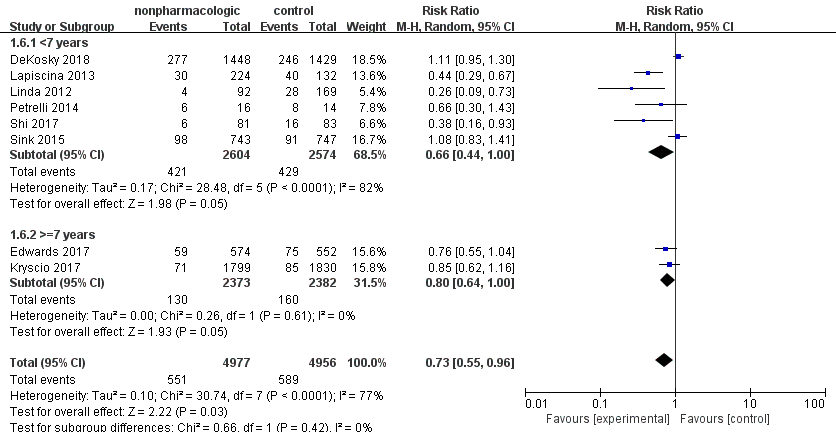
**

1. Forest plot of subgroups by the duration of follow-up(< 7 years and ≥ 7 years)

**Fig. S3** Forest plot of subgroups

1. Forest plot of subgroups by the number of sample size(< 500 and ≥ 500)
2. Forest plot of subgroups by the area of RCTs(America, Asia and Europe)
3. Forest plot of subgroups by the type of nonpharmacological interventions(cognitive training,exercise and dietary)
4. Forest plot of subgroups by the duration of follow-up(< 7 years and ≥ 7 years)
