# Supplementary material for: Do nonpharmacological interventions prevent cognitive decline? a systematic review and meta-analysis
Source: Transl Psychiatry. 2020 Jan 21;10:19. doi: 10.1038/s41398-020-0690-4 (PMC7026127; doi:10.1038/s41398-020-0690-4)
Supplement: Supplementary file 7 — Fig. S5 [file 41398_2020_690_MOESM7_ESM.doc]

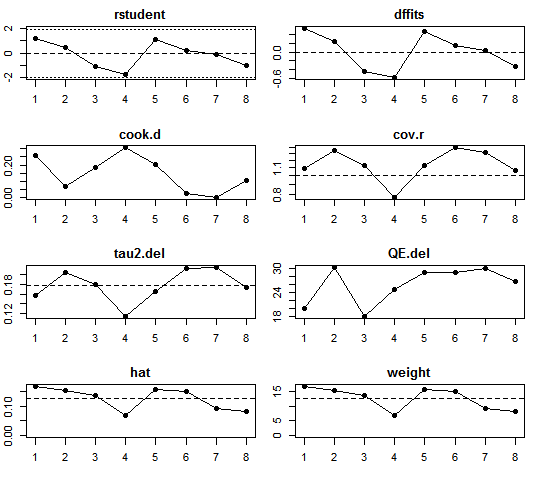


**(A) Influence Analyses**


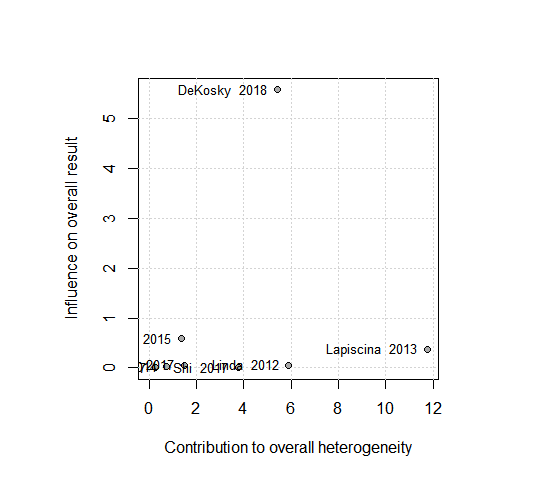


1. **Baujat Plot**


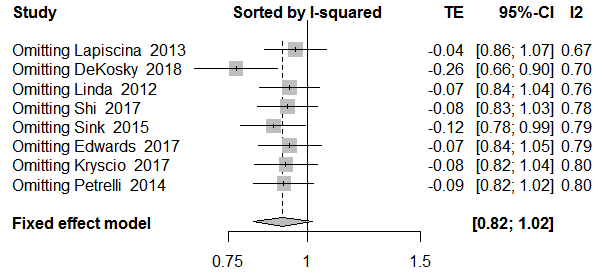


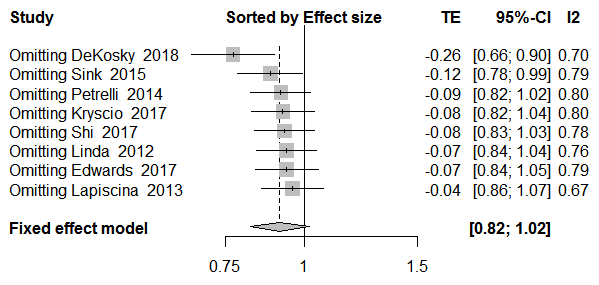


**(C) Leave-One-Out-Analyses**

**Fig. S5 Outlier and influence analysis**

**(A) Influence Analyses**

X-axis represents 8 trials that reported the incidence of MCI or dementia

Y-axis

**rstudent:** The studentized deleted residual for a particular study formalizes a proper outlier test under a mean shift outlier model.

**dffits:** The DIFFITS value of a study indicates in standard deviations how much the predicted pooled effect changes after excluding this study.

**cook.d:** The Cook’s distance resembles the Mahalanobis distance you may know from outlier detection in conventional multivariate statistics. It is the distance between the value once the study is included compared to when it is excluded.

**cov.r:** The covariance ratio is the determinant of the variance-covariance matrix of the parameter estimates when the study is removed, divided by the determinant of the variance-covariance matrix of the parameter estimates when the full dataset is considered. Importantly, values of cov.r < 1 indicate that removing the study will lead to a more precise effect size estimation (i.e., less heterogeneity).

**tau2.del:** It is one type of heterogeneity measures.

**QE:** QE is the statistic of the homogeneity test

**hat:** hat values.

1. **Baujat Plot**

**(C) Leave-One-Out-Analyses**
