# Supplementary material for: Do nonpharmacological interventions prevent cognitive decline? a systematic review and meta-analysis
Source: Transl Psychiatry. 2020 Jan 21;10:19. doi: 10.1038/s41398-020-0690-4 (PMC7026127; doi:10.1038/s41398-020-0690-4)
Supplement: Supplementary file 8 — Fig. S6 [file 41398_2020_690_MOESM8_ESM.doc]

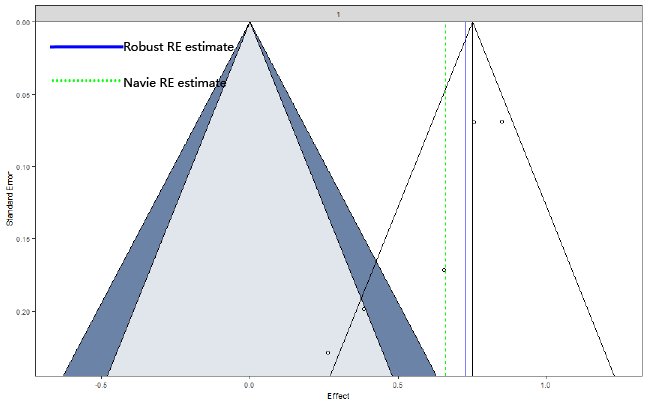


**(A) Contour-enhanced funnel plot of observed nonpharmacological interventions effects from included randomized clinical trials that reported the incidence of MCI or dementia.**


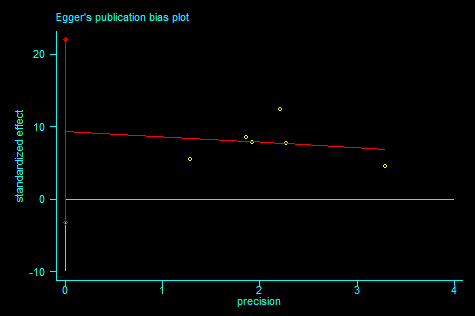


**(B) Egger’s publication bias plot**

**Fig. S6 Funnel plot and Egger test**

**(A) Contour-enhanced funnel plot of observed nonpharmacological interventions effects from included randomized clinical trials that reported the incidence of MCI or dementia**

**(B) Egger’s publication bias plot**
