# Supplementary material for: Do nonpharmacological interventions prevent cognitive decline? a systematic review and meta-analysis
Source: Transl Psychiatry. 2020 Jan 21;10:19. doi: 10.1038/s41398-020-0690-4 (PMC7026127; doi:10.1038/s41398-020-0690-4)
Supplement: Supplementary file 9 — Table S1 [file 41398_2020_690_MOESM9_ESM.doc]

**Table S1.** Diagnosis of MCI or dementia in the included trials

| First author | Authors’ judgement | How to diagnose MCI or dementia |
| --- | --- | --- |
| Linda 2012 | High risk | 1. Progression to dementia: Clinical diagnosis of dementia was made with the DSM-IV criteria. The primary outcome indicator was the rate of “conversion” to clinical dementia using DSM-IV criteria compared between I and C groups after 1 year of intervention. Staging of dementia was evaluated by the CDR, a semistructured interview of 6 dimensions, including memory,orientation, judgment, community affairs, hobbies and habits, and personal care. A CDR of 1 to 3 indicates mild to severe dementia.  2. Cognitive test scores: Differences in the change in cognitive scores from baseline between the I and C groups was considered as cognitive outcome indicator. The Cantonese version of the ADAS-Cog, digit span, delay recall, category verbal fluency tests, trail making, and Mini-Mental State Examination were assessed. |
| Kryscio 2017 | Low risk | Dementia case ascertainment relied on a consensus review of the cognitive screens and medical records for men with suspected dementia who visited their physician for an evaluation or by review of all available information, including a functional assessment screen. Dementia incidence, the primary end point, was determined by 1 of 2 methods. First, if participants failed both the first tier of the screen (MIS score ≤ 5 of 8) and the second tier (T Score ≤ 35 on the CERAD battery; total score ≤35 on the TICS-m), then they were encouraged to obtain a memory workup from their local clinician and share medical records with PREADViSE trial investigators. Medical records were reviewed by a team of 2 to 3 expert neurologists and 2 to 3 expert neuropsychologists to determine consensus diagnoses. Participants who did not obtain the workup were assessed by additional longitudinal measures collected during the study. These included the Ascertain Dementia 8-Item Informant Questionnaire (AD 8) Dementia Screening Interview, self-reported medical history, self-reported medication use, and cognitive scores, including the MIS, CERAD T Score, New York University Paragraph Delayed Recall, and TICS-m. An AD 8 of 1 or greater (at any time during follow-up) as well as a self-reported dementia diagnosis, use of amemory-enhancing prescription drug (eg, donepezil, rivastigmine, galantamine, ormemantine), or cognitive score 1.5 SDs or more below expected performance yielded a dementia diagnosis. The diagnosis date was assigned to the earliest event. |
| Petrelli 2014 | High risk | Definition of MCI:  Subjective cognitive impairment or impairment recognized by the clinician and largely intact activities of daily living according to medical history Neuropsychological test battery, scores of ≤1.5 SD below the population norm mean defined as impaired  Attention: two subtests (numbers and letters) of the Brief Test of Attention.  Memory: DemTect word list (direct and delayed recall) and the delayed recall of the Rey Complex Figure Test.  Executive functions: DemTect working memory and DemTect semantic verbal fluency task(‘supermarket’) and letter verbal fluency task (controlled oral word association – FAS).  Visuo-construction: copy tasks of the Rey Complex Figure Test and pentagon drawing of the MMSE.  Language: MMSE language items. |
| Sink 2015 | Low risk | Participants who scored 88 points or less on the 3MSE were sent for central adjudication by a panel (blinded to treatment assignment) of 8 clinical experts in the diagnosis of late-life cognitive impairment. Each case was assigned to 2 independent adjudicators; disagreements were resolved by the full panel. Based on 2011 criteria from the National Institute on Aging and the Alzheimer’s Association, MCI and dementia were adjudicated. |
| Lapiscina 2013 | Low risk | Medical records of all participants were checked to collect incidence events including mild cognitive impairment (MCI), dementia and depression. This information was sent to The Adjudication Committee. This Committee reviewed the suitability of diagnoses according to the available information. Additionally, participants with a pathological cognitive screening test were re-evaluated by a neurologist to determine the presence or absence of MCI or dementia. Diagnoses of dementia or MCI from the Adjudication Committee were based on assessments recorded in clinical records and usually made by neurologists upon the request of either general practitioners or participants. In addition, we performed a comprehensive cognitive evaluation to identify MCI or dementia. The use of both methods (review of medical record based on referrals by general practitioners and personalised comprehensive neuropsychiatric assessment) are likely to have increased the sensitivity in detecting MCI and dementia. |
| Edwards 2017 | Low risk | Defined dementia as the first occurrence of any of the following:  1. Cognitive and functional impairment defined as follows: a) memory composite score at or below -1.5 SD of the baseline sample mean and reasoning composite, speed composite, or vocabulary score at or below -1.5 SD of the baseline mean (for assessment details see [Jobe JB, Smith DM, Ball KK, Tennstedt SL, Marsiske M, Willis SL,et al. ACTIVE: A cognitive intervention trial to promote independence in older adults. Control Clin Trials 2001;22:453–79]), and b) MDS IADL total score at or below the 10th percentile of the baseline (self-reported).  2. A score of ,22 on the MMSE, with all subsequent MMSE assessments at ,22 or missing [McDowell I, Kristjansson B, Hill GB, Hebert R. Community screening for dementia: The Mini Mental State Exam (MMSE) and modified Mini-Mental State Exam (3MS) compared. J Clin Epidemiol 1997;50:377–83].  3. Self- or proxy-report of diagnosis of dementia or Alzheimer’s disease during the follow-up. |
| Shi 2017 | High risk | According to Diagnostic and Statistical Manual of Mental Disorders of American Psychiatric Association (DSM IV) [Anxiety disorders in the fourth edition of the classification of mental disorders prepared by the American psychiatric association: diagnostic and statistical manual of mental disorders (DMS-IV-ptions book]. Psychiatr Pol 1994; 28: 255-68]. |
| DeKosky 2018 | Low risk | The panel consisted of 2 neurologists with expertise in dementia diagnosis; 2 neuropsychologists experienced in cognitive assessment of dementia; and a psychometrician with extensive experience in training, administration, and scoring of the CDR. Participants classified as reaching dementia end point at this point were then referred for a full neurological evaluation and a magnetic resonance imaging(MRI) scan at the clinical site to confirm that the participant met clinical criteria for dementia and assess for atypical causes of dementia. The MRIs were reviewed according to a standard protocol by 2 boardcertified neuroradiologists, also blinded to treatment assignment, and ratings were given for cortical atrophy; ventricular size; subcortical white matter lesions; and presence, size, and number of brain infarcts. These data and the neuroradiologists’ clinical readings were available to the adjudication panel in its diagnostic decision-making, and the adjudicating neurologists also reviewed the scans themselves as part of their diagnostic process. |
