# Supplementary material for: Do nonpharmacological interventions prevent cognitive decline? a systematic review and meta-analysis
Source: Transl Psychiatry. 2020 Jan 21;10:19. doi: 10.1038/s41398-020-0690-4 (PMC7026127; doi:10.1038/s41398-020-0690-4)
Supplement: Supplementary file 10 — Table S2 [file 41398_2020_690_MOESM10_ESM.doc]

**Table S2. Summary of Strength of Evidence for Outcomes**

| **Certainty assessment** | | | | | | | **№ of patients** | | **Effect** | | **Certainty** | **Importance** |
| --- | --- | --- | --- | --- | --- | --- | --- | --- | --- | --- | --- | --- |
| **№ of studies** | **Study design** | **Risk of bias** | **Inconsistency** | **Indirectness** | **Imprecision** | **Other considerations** | **Nonpharmacologic** | **Control** | **Relative (95% CI)** | **Absolute (95% CI)** |
| **the incidence of MCI or dementia** | | | | | | | | | | | | |
| 8 | randomised trials | not serious | serious ^a^ | not serious | not serious | none | 551/4977 (11.1%) | 589/4956 (11.9%) | **RR 0.73** (0.55 to 0.96) | **32 fewer per 1,000** (from 53 fewer to 5 fewer) | ⨁⨁⨁◯ MODERATE | CRITICAL |
| **ADAS-Cog** | | | | | | | | | | | | |
| 2 | randomised trials | serious ^b^ | very serious ^a,c^ | not serious | very serious ^d^ | none | 82 | 82 | - | MD **0.69 lower** (1.52 lower to 0.14 higher) | ⨁◯◯◯ VERY LOW | CRITICAL |
| **ADL** | | | | | | | | | | | | |
| 2 | randomised trials | serious ^b^ | not serious | not serious | serious ^e^ | none | 122 | 134 | - | MD **0.73 higher** (0.65 higher to 0.8 higher) | ⨁⨁◯◯ LOW | IMPORTANT |
| **MMSE** | | | | | | | | | | | | |
| 9 | randomised trials | not serious | serious ^a,c^ | not serious | serious ^c^ | none | 1168 | 1140 | - | MD **0.04 higher** (0.04 lower to 0.12 higher) | ⨁⨁◯◯ LOW | IMPORTANT |
| **MMSE(ΔE vs. ΔC)** | | | | | | | | | | | | |
| 6 | randomised trials | not serious | serious ^a^ | not serious | not serious | none | 763 | 743 | - | MD **0.5 higher** (0.46 higher to 0.53 higher) | ⨁⨁⨁◯ MODERATE | IMPORTANT |
| **GDS** | | | | | | | | | | | | |
| 3 | randomised trials | serious ^b^ | very serious ^a,c^ | not serious | very serious ^d^ | none | 148 | 155 | - | MD **0.36 lower** (0.45 lower to 0.26 lower) | ⨁◯◯◯ VERY LOW | IMPORTANT |

**CI:** Confidence interval; **RR:** Relative risk; **MD:** Mean difference

#### Explanations

a. Downgraded one level for heterogeneity : the statistical test for heterogeneity showed that large variation (I² >50%) existed in point estimates due to among-study differences.

b. Downgraded one level for risk of bias: most of the included RCTs had unclear risk of concealment of allocation

c. 95% Confidence intervals(CIs) around the pooled included no effect and appreciable benefit

d. Downgraded two levels for imprecision: the sample size was less than 300, the number of events not high, 95% Confidence intervals(CIs) show overlap and 95% Confidence intervals(CIs) crossed the line of no effect and appreciable benefit

e. Downgraded one level: total sample size is lower than 300
